# Inflammasome signaling is dispensable for ß-amyloid-induced neuropathology in preclinical models of Alzheimer’s disease

**DOI:** 10.3389/fimmu.2024.1323409

**Published:** 2024-01-29

**Authors:** Sahana Srinivasan, Daliya Kancheva, Sofie De Ren, Takashi Saito, Maude Jans, Fleur Boone, Charysse Vandendriessche, Ine Paesmans, Hervé Maurin, Roosmarijn E. Vandenbroucke, Esther Hoste, Sofie Voet, Isabelle Scheyltjens, Benjamin Pavie, Saskia Lippens, Marius Schwabenland, Marco Prinz, Takaomi Saido, Astrid Bottelbergs, Kiavash Movahedi, Mohamed Lamkanfi, Geert van Loo

**Affiliations:** ^1^ VIB Center for Inflammation Research, Ghent, Belgium; ^2^ Department of Biomedical Molecular Biology, Ghent University, Ghent, Belgium; ^3^ Brain and Systems Immunology Lab, Brussels Center for Immunology, Vrije Universiteit Brussel, Brussels, Belgium; ^4^ Neuroscience Therapeutic Area, Janssen Research and Development, Beerse, Belgium; ^5^ Laboratory for Proteolytic Neuroscience, RIKEN Center for Brain Science, Saitama, Japan; ^6^ Department of Neurocognitive Science, Institute of Brain Science, Nagoya City University Graduate School of Medical Sciences, Nagoya, Aichi, Japan; ^7^ Department of Neuroscience and Pathobiology, Research Institute of Environmental Medicine, Nagoya University, Nagoya, Aichi, Japan; ^8^ VIB Bioimaging Core, Ghent, Belgium; ^9^ Institute of Neuropathology Medical Center, University of Freiburg, Freiburg, Germany; ^10^ Signalling Research Centres BIOSS and CIBSS, University of Freiburg, Freiburg, Germany; ^11^ Center for Basics in NeuroModulation (NeuroModulBasics), Faculty of Medicine, University of Freiburg, Freiburg, Germany; ^12^ Department of Internal Medicine and Pediatrics, Ghent University, Ghent, Belgium

**Keywords:** Alzheimer’s disease, ß-amyloid, microglia, neuroinflammation, inflammasome

## Abstract

**Background:**

Alzheimer’s disease (AD) is the most common neurodegenerative disorder affecting memory and cognition. The disease is accompanied by an abnormal deposition of ß-amyloid plaques in the brain that contributes to neurodegeneration and is known to induce glial inflammation. Studies in the *APP/PS1* mouse model of ß-amyloid-induced neuropathology have suggested a role for inflammasome activation in ß-amyloid-induced neuroinflammation and neuropathology.

**Methods:**

Here, we evaluated the *in vivo* role of microglia-selective and full body inflammasome signalling in several mouse models of ß-amyloid-induced AD neuropathology.

**Results:**

Microglia-specific deletion of the inflammasome regulator A20 and inflammasome effector protease caspase-1 in the *App^NL-G-F^* and *APP/PS1* models failed to identify a prominent role for microglial inflammasome signalling in ß-amyloid-induced neuropathology. Moreover, global inflammasome inactivation through respectively full body deletion of caspases 1 and 11 in *App^NL-G-F^* mice and Nlrp3 deletion in *APP/PS1* mice also failed to modulate amyloid pathology and disease progression. In agreement, single-cell RNA sequencing did not reveal an important role for Nlrp3 signalling in driving microglial activation and the transition into disease-associated states, both during homeostasis and upon amyloid pathology.

**Conclusion:**

Collectively, these results question a generalizable role for inflammasome activation in preclinical amyloid-only models of neuroinflammation.

## Introduction

Neurodegeneration is a complex, multifaceted process that degrades neuronal structure and function, ultimately leading to cognitive and/or motor disability and dementia (1). Alzheimer’s disease (AD) is the most common form of neurodegenerative dementia, and currently affects more than 30 million people over the age of 65 worldwide (2). Neuronal damage and subsequent neurodegeneration in AD are associated with the accumulation of β-amyloid (Aβ) in extracellular plaques and the intracellular aggregation of hyperphosphorylated tau in neurofibrillary tangles (2). For a long time the focus has been on cell-autonomous processes primarily affecting neurons, whereas glial involvement is progressively taking center stage as an important mediator of neurodegenerative pathology in AD (3).

Microglia are CNS resident macrophages that are distributed across the brain parenchyma (4–6). When activated by damage-associated or pathogen-associated molecular patterns (DAMPs and PAMPs, respectively), microglia rapidly move toward the site of injury to initiate an innate immune response in order to cope with the insult (6, 7). In AD, Aβ oligomers that are generated by cleavage of amyloid precursor protein (APP) and amyloid fibrils in the amyloid plaques, bind various microglial cell-surface receptors, which prompt microglial activation and release of inflammatory mediators (8–10). Some of the most robust evidence for microglial involvement in AD came from the identification of AD risk genes in genome-wide association studies (11–19), suggesting that microglia are not merely bystanders, but possibly causally involved in AD pathogenesis. However, the role of specific inflammatory pathways and mechanisms of microglial activation in Aβ-induced neuroinflammation and neurodegenerative pathology require further analysis.

The NF-κB signaling pathway critically regulates inflammatory responses and has been linked to the pathogenesis of several neurodegenerative disorders (20–22). Though baseline activity is very low in glia, NF-κB activity is significantly increased in glia surrounding Aβ plaques (23). Increased levels of the NF-κB p65 subunit are reported in cortical neurons and glia of AD patients (24). This in turn upregulates transcription of pro-inflammatory target genes that promote neuroinflammation (25), as well as that of BACE1, which promotes APP cleavage to generate additional Aβ (26). NF-κB activation is also a prerequisite for priming the NLR family pyrin domain containing 3 (NLRP3) inflammasome, a cytosolic multimeric protein complex that recruits and activates procaspase-1, a protease that subsequently cleaves and releases the pro-inflammatory cytokines interleukin (IL)-1β and IL-18 and drives an inflammatory form of cell death known as pyroptosis by cleaving gasdermin D (GSDMD) (27). Increased levels of NLRP3, caspase-1, IL-1β, IL-18, the inflammasome adaptor protein ASC and cleaved GSDMD have been reported in reactive microglia that surround Aβ plaques in brain of AD patients (28–31). Moreover, *in vitro* studies have demonstrated that fibrillar Aβ stimulates microglia activation and IL-1β production via assembly of the NLRP3 inflammasome and caspase-1 activation (32). Finally, an *in vivo* role for the NLRP3/caspase-1 inflammasome axis was subsequently proposed in the *APP/PS1* model of Aβ-induced neuropathology (33). However, the relative significance of microglial NLRP3 inflammasome activation in additional *in vivo* models of Aβ-induced neuropathology has not been explored.

Here, we investigated the role of microglial and full body inflammasome involvement in the *App^NL-G-F^* and *APP/PS1* mouse models of ß-amyloid-induced neuropathology. *APP/PS1* mice ectopically overexpress the KM670/671NL ‘Swedish’ mutated amyloid precursor protein (APP) concomitant with mutant human PS1 (presenilin-1) in CNS neurons, and develop an early and robust AD pathology (34). In contrast, *App^NL-G-F^* mice express humanized sequences and clinical mutations in the endogenous mouse *App* gene locus such that Aβ42 is produced without APP overexpression (35). As a result, Aβ-induced pathophysiological readouts develop more slowly in *App^NL-G-F^* mice compared to aggressive disease progression in *APP* transgenic mice (36). First, we hypothesized that microglial deletion of A20, a negative regulator of NF-κB signaling that controls microglia inflammasome activation and CNS inflammation (37), may exacerbate ß-amyloid-induced neuroinflammation and AD outcomes (**Supplementary Figure 1A**). Secondly, we tested the assumption that microglial deletion of the central inflammasome effector protease caspase-1 may suppress microglia activation, neuroinflammation and amyloid pathology (**Supplementary Figure 1B**). Unexpectedly, our results in the *App^NL-G-F^* and *APP/PS1* mouse models argue against a significant role for microglial inflammasome activation in Aß-associated neuroinflammation and neurodegenerative pathology. Further supporting these hypotheses, we found that full body deletion of caspase-1 in *App^NL-G-F^* mice (**Supplementary Figure 1B**) and full body Nlrp3 deletion in *APP/PS1* mice (**Supplementary Figure 1C**) both failed to inhibit ß-amyloid-induced gliosis, inflammation and plaque burden in diseased mice. Consistent herewith, single-cell gene expression profiling revealed only minor transcriptional changes in microglia from Nlrp3-deficient *APP/PS1* mice, negating inflammasome signaling as a driver of microglial cell states during amyloid pathology. Taken together, these findings question a generalizable role for inflammasome activation in mouse models of ß-amyloid-induced neuroinflammation and pathology.

## Results

### Microglial A20 deletion promotes CNS hyperinflammation but does not increase ß-amyloid-induced pathology in *App^NL-G-F^* mice

A20 acts as a key inhibitor of NF-κB signaling (38), and its deficiency leads to increased expression of inflammatory mediators in microglia (37, 39). Additionally, previous research has demonstrated that A20 acts as a negative regulator in priming and activation of the Nlrp3 inflammasome in microglia (37). To investigate the impact of microglial NF-κB and Nlrp3 inflammasome hyperactivation in Aß-associated neuroinflammation, we crossed mice carrying floxed alleles for *A20* (40) to *Cx3cr1* ^CreErt2^ knock-in mice to allow Cre recombinase-mediated long-term A20 deletion in microglia following tamoxifen (TAM) treatment (41). Control mice (*A20^FL^*, expressing A20) and mice with tamoxifen-inducible A20 deficiency in microglia (*A20^FL/FL^ Cx3cr1^CreErt2^*, hereafter A20^Cx3cr1-KO^) were first crossed to *App^NL-G-F^* animals expressing mutant APP from the endogenous locus (35). At 4-6 weeks of age, the progeny was subcutaneously injected with TAM to activate Cre recombinase and generate *A20^FL^App^NL-G-F^* mice and *A20^Cx3Cr1-KO^App^NL-G-F^* mice which do (*A20^FL^)* or do not (*A20^CxcCr1-KO^*) express A20 in microglia in an amyloid model of AD pathology. These mice were compared with age-matched *App^WT^* mice to accurately assign differences emerging from the mutant APP genetic background or A20 genotype, and possible interaction effects (**Supplementary Figures 2A–C**).

Successful downregulation of A20 in microglia was confirmed at the protein level by Western blotting of *ex vivo* isolated and fluorescence-activated cell sorted (FACS) microglia (**Supplementary Figure 2D**), and of primary microglia cultured *in vitro* (**Supplementary Figure 2E**). Consistent with enhanced NF-κB activation in A20 deficient conditions, qPCR analysis confirmed that A20 deletion results in increased expression of the pro-inflammatory cytokines and chemokines *Il1β, Il6, Tnf*, *Ccl2* and *Ccl5* in hippocampal lysates of A20^Cx3cr1-KO^ mice when compared to A20^FL^ mice (**Supplementary Figure 3A**). These differences in expression persisted for *Ccl5*, *Ccl2*, and *Tnf* in aged (56 week-old) animals (**Supplementary Figure 3B**). However, expression levels of these A20-modulated cytokines and chemokines were not majorly different in A20^Cx3Cr1-KO^ *App^NL-G-F^* mice and A20^Cx3Cr1-KO^ mice in a non-AD *App^WT^* genetic background (**Supplementary Figures 3A–B**). This suggests that A20 deletion in microglia promotes inflammatory cytokine production regardless of the mutant APP allele.

**Figure 1:**
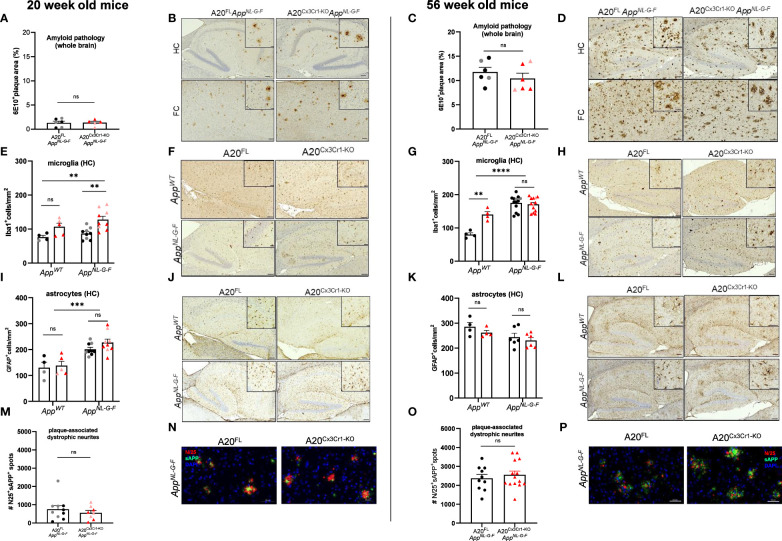
Microglial A20 deficiency does not exacerbate AD pathology in *App^NL-G-F^* mice. **(A)** Quantification of the area covered by 6E10^+^ amyloid plaque deposits in whole brains of 20 week-old A20^FL^ (black) and A20^Cx3Cr1-KO^ *App^NL-G-F^* mice (red). Each symbol represents one mouse, n=5-6 per group (males, dark color; females, pale color). Data are represented as mean ± SEM. **(B)** Immunohistochemistry for 6E10^+^ amyloid plaque load in the hippocampus (HC) and frontal cortex (FC) of 20 week-old A20^FL^ and A20^Cx3Cr1-KO^ *App^NL-G-F^* mice. Scale bars: 100 µm (inset: 50 µm). Representative images are displayed. **(C)** Quantification of the number of 6E10^+^ amyloid plaque deposits in whole brains of 56 week-old A20^FL^ (black) and A20^Cx3Cr1-KO^ *App^NL-G-F^* mice (red). Each symbol represents one mouse, n=6 per group (males, dark color; females, pale color). Data are represented as mean ± SEM. **(D)** Immunohistochemistry for 6E10^+^ amyloid plaque load in the hippocampus (HC) and frontal cortex (FC) of 56 week-old A20^FL^ and A20^Cx3Cr1-KO^ *App^NL-G-F^* mice. Scale bars: 100 µm (inset: 50 µm). Representative images are displayed. **(E)** Quantification of the number of Iba1^+^ microglia in the hippocampus of 20 week-old A20^FL^ and A20^Cx3Cr1-KO^ *App^WT^* and *App^NL-G-F^* mice. Each symbol represents one mouse, n=4-5 per group (*App^WT^*); n=9 per group (*App^NL-G-F^*). Data are represented as mean ± SEM. Significant differences are determined using two-way ANOVA (**, p<0.001). **(F)** Immunohistochemistry for Iba1^+^ microglia in the hippocampus of 20 week-old A20^FL^ and A20^Cx3Cr1-KO^ *App^WT^* and *App^NL-G-F^* mice. Scale bars: 100µm (inset: 50 µm). Representative images are displayed. **(G)** Quantification of the number of Iba1^+^ microglia in the hippocampus of 56 week-old A20^FL^ and A20^Cx3Cr1-KO^ *App^WT^* and *App^NL-G-F^* mice. Each symbol represents one mouse, n=4 per group (*App^WT^*); n=13 per group (*App^NL-G-F^*). Data are represented as mean ± SEM. Significant differences are determined using two-way ANOVA (**p<0.001; ****p<0.0001). **(H)** Immunohistochemistry for Iba1^+^ microglia in the hippocampus of 56 week-old A20^FL^ and A20^Cx3Cr1-KO^ *App^WT^* and *App^NL-G-F^* mice. Scale bars: 100µm (inset: 50 µm). Representative images are displayed. **(I)** Quantification of the number of GFAP^+^ astrocytes in the hippocampus of 20 week-old A20^FL^ and A20^Cx3Cr1-KO^ *App^WT^* and *App^NL-G-F^* mice. Each symbol represents one mouse, n=4-5 per group (*App^WT^*); n=9 per group (*App^NL-G-F^*). Data are represented as mean ± SEM. Significant differences are determined using two-way ANOVA (***p<0.001). **(J)** Immunohistochemistry for GFAP^+^ astrocytes in the hippocampus of 20 week-old A20^FL^ and A20^Cx3Cr1-KO^ *App^WT^* and *App^NL-G-F^* mice. Scale bars: 100µm (inset: 50 µm). Representative images are displayed. **(K)** Quantification of the number of GFAP^+^ astrocytes in the hippocampus of 56 week-old A20^FL^ and A20^Cx3Cr1-KO^ *App^WT^* and *App^NL-G-F^* mice. Each symbol represents one mouse, n=4 per group (*App^WT^*); n=6 per group (*App^NL-G-F^*). Data are represented as mean ± SEM. **(L)** Immunohistochemistry for GFAP^+^ astrocytes in the hippocampus of 56 week-old A20^FL^ and A20^Cx3Cr1-KO^ *App^WT^* and *App^NL-G-F^* mice. Scale bars: 100µm (inset: 50 µm). Representative images are displayed. **(M)** Quantification of the number of plaque-associated dystrophic neurites (N25^+^ sAPP^+^) in whole brains of 20 week-old A20^FL^ and A20^Cx3Cr1-KO^ *App^NL-G-F^* mice. Each symbol represents one mouse, n=8-10 per group. Data are represented as mean ± SEM. **(N)** Immunohistochemistry for N25^+^ sAPP^+^ plaque-associated dystrophic neurites in whole brains of 20-week-old A20^FL^ and A20^Cx3Cr1-KO^ *App^NL-G-F^* mice. Scale bars: 50 µm. Representative images are displayed. **(O)** Quantification of the number of plaque-associated dystrophic neurites (N25^+^ sAPP^+^) in whole brains of 56 week-old A20^FL^ and A20^Cx3Cr1-KO^ *App^NL-G-F^* mice. Each symbol represents one mouse, n=10-14 per group. Data are represented as mean ± SEM. **(P)** Immunohistochemistry for N25^+^ sAPP^+^ plaque-associated dystrophic neurites in whole brains of 56 week-old A20^FL^ and A20^Cx3Cr1-KO^ *App^NL-G-F^* mice. Scale bars: 50 µm. Representative images are displayed. ns, not significant.

We next assessed brain pathology in A20-deficient *App^NL-G-F^* animals at the histological level. Brain sections of A20^FL^ *App^NL-G-F^* and A20^Cx3Cr1-KO^ *App^NL-G-F^* mice, as well as age-matched *App^WT^* controls were evaluated for differences in amyloid plaque burden, microgliosis and astrogliosis, as well as for differences in plaque-associated axonal damage. As expected, brains of *App^WT^* control animals were devoid of amyloid deposits, whereas amyloid plaques were readily visualized by anti-Aβ antibody staining in brain sections of *App^NL-G-F^* animals (**Figures 1A, B** and **Supplementary Figure 4**). However, microglial deletion of A20 did not significantly alter amyloid plaque deposition in brains of young (20 weeks of age, **Figures 1A, B** and **Supplementary Figures 4A–F**) or aged (56 weeks of age, **Figures 1C, D** and **Supplementary Figures 4G–L**) *App^NL-G-F^* animals, suggesting that hyperactive NF-κB and inflammasome signaling by long-term A20 deletion in microglia may not be a critical modulator of Aβ plaque formation in *App^NL-G-F^* animals.

Chronic Aβ deposition in brain parenchyma also induces chronic glial activation, characterized by higher microglia proliferation and changes in microglia morphology (42). Moreover, microglial A20 deletion is associated with a significant increase in microglia proliferation in non-AD mouse models of neuroinflammation (37). In agreement, we observed an increased number of hippocampal Iba1^+^ microglia in brains of 20 week-old A20^Cx3Cr1-KO^ mice compared to A20^FL^ mice, which was even more pronounced in the *App^NL-G-F^* genetic background (**Figures 1E, F** and **Supplementary Figures 5A, B**). Aged (56 week-old) *App^NL-G-F^* mice displayed increased levels of hippocampal microglia proliferation compared to mice on the *App^WT^* background, regardless whether they had A20^FL^ or A20^Cx3Cr1-KO^ alleles, confirming that the mutant APP genetic background on itself boosts microglial proliferation (**Figures 1G, H** and **Supplementary Figures 5C, D**).

In contrast to the microgliosis, hippocampal astrocyte numbers were unaffected by microglial deletion of A20. The number of GFAP^+^ hippocampal astrocytes was significantly increased in young *App^NL-G-F^* mice compared to *App^WT^* mice, which likely was a consequence of early Aβ-induced brain pathology (**Figures 1I, J** and **Supplementary Figures 5E, F**). However, the number of GFAP+ hippocampal astrocytes reached similar levels across all experimental groups at older age (**Figures 1K, L** and **Supplementary Figures 5G, H**).

**Figure 2:**
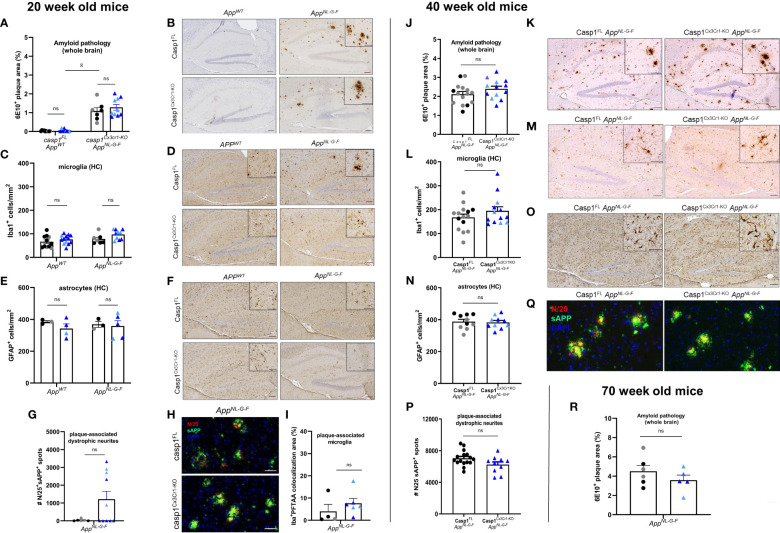
Microglial casp1 deficiency does not suppress AD pathology in *App^NL-G-F^* mice. **(A)** Quantification of 6E10^+^ amyloid plaque pathology over whole brains of 20 week-old casp1^FL^ (black) and casp1^Cx3Cr1-KO^ (blue) *App^WT^* and *App^NL-G-F^* mice. Each symbol represents one mouse, n=7-10 (males, dark color; females, pale color). Data are represented as mean ± SEM. Significant differences are determined using two-way ANOVA (*, p < 0.05). **(B)** Immunohistochemistry for 6E10^+^ amyloid plaques in hippocampus of 20 week-old casp1^FL^ and casp1^Cx3Cr1-KO^ *App^WT^* and *App^NL-G-F^* mice. Scale bars: 100 µm (inset: 50 µm). Representative images are displayed. **(C)** Quantification of the number of hippocampal Iba1^+^ microglia in 20 week-old casp1^FL^ and casp1^Cx3Cr1-KO^ *App^WT^* and *App^NL-G-F^* mice. Each symbol represents one mouse, n=7-13 (*App^WT^*), n=7-10 (*App^NL-G-F^*). Data are represented as mean ± SEM. **(D)** Immunohistochemistry for Iba-1^+^ microglia in the hippocampus of 20 week-old casp1^FL^ and casp1^Cx3Cr1-KO^ *App^WT^* and *App^NL-G-F^* mice. Scale bars: 100 µm (inset: 50 µm). Representative images are displayed. **(E)** Quantification of the number of hippocampal GFAP^+^ astrocytes in 20 week-old casp1^FL^ and casp1^Cx3Cr1-KO^ *App^WT^* and *App^NL-G-F^* mice. Each symbol represents one mouse, n=3-5 per group. Data are represented as mean ± SEM. **(F)** Immunohistochemistry for GFAP^+^ astrocytes in the hippocampus of 20 week-old casp1^FL^ and casp1^Cx3Cr1-KO^ *App^WT^* and *App^NL-G-F^* mice. Scale bars: 100 µm (inset: 50 µm). Representative images are displayed. **(G)** Quantification of the number of plaque-associated dystrophic neurites (N25^+^ sAPP^+^) in 20 week-old casp1^FL^ and casp1^Cx3Cr1-KO^ *App^NL-G-F^* mice. Each symbol represents one mouse, n=6-10 per group. Data are represented as mean ± SEM. **(H)** Immunohistochemistry for N25^+^ sAPP^+^ plaque-associated dystrophic neurites in the brain parenchyma of 20 week-old casp1^FL^ and casp1^Cx3Cr1-KO^ *App^NL-G-F^* mice. Scale bars: 50 µm. Representative images are displayed. **(I)** Quantification of the number of plaque-associated microglia, measured as percentage Iba^+^ PFTAA colocalization area, in 20 week-old casp1^FL^ and casp1^Cx3Cr1-KO^ *App^NL-G-F^* mice. Each symbol represents one mouse, n=4-6 per group. Data are represented as mean ± SEM. **(J)** Quantification of 6E10^+^ amyloid plaque pathology over whole brains of 40 week-old casp1^FL^ (black) and casp1^Cx3Cr1-KO^ *App^NL-G-F^* (blue) mice. Each symbol represents one mouse, n=13-15 per group (males, dark color; females, pale color). Data are represented as mean ± SEM. **(K)** Immunohistochemistry for 6E10^+^ amyloid plaque load in hippocampus of 40 week-old casp1^FL^ and casp1^Cx3Cr1-KO^ *App^NL-G-F^* mice. Scale bars: 100 µm (inset: 50µm). Representative images are displayed. **(L)** Quantification of the number of hippocampal Iba1^+^ microglia in 40 week-old casp1^FL^ and casp1^Cx3Cr1-KO^ *App^NL-G-F^* mice. Each symbol represents one mouse, n=13-15. Data are represented as mean ± SEM. **(M)** Immunohistochemistry for Iba-1^+^ microglia in the hippocampus of 40 week-old casp1^FL^ and casp1^Cx3Cr1-KO^ *App^NL-G-F^* mice. Scale bars: 100 µm (inset: 50µm). Representative images are displayed. **(N)** Quantification of the number of hippocampal GFAP^+^ astrocytes in 40 week-old casp1^FL^ and casp1^Cx3Cr1-KO^ *App^NL-G-F^* mice. Each symbol represents one mouse, n=10. Data are represented as mean ± SEM. **(O)** Immunohistochemistry for GFAP^+^ astrocytes in hippocampus of 40 week-old casp1^FL^ and casp1^Cx3Cr1-KO^ *App^NL-G-F^* mice. Scale bars: 100 µm (insets 50µm). Representative images are displayed. **(P)** Quantification of the number of plaque-associated dystrophic neurites (N25^+^ sAPP^+^) in 40 week-old casp1^FL^ and casp1^Cx3Cr1-KO^ *App^NL-G-F^*. Each symbol represents one mouse. Data are represented as mean ± SEM. **(Q)** Immunohistochemistry for N25^+^ sAPP^+^ dystrophic neurites in brain parenchyma of 40 week-old casp1^FL^ and casp1^Cx3Cr1-KO^ *App^NL-G-F^* mice. Scale bars: 50 µm. Representative images are displayed. **(R)** Quantification of 6E10^+^ amyloid plaque pathology over whole brains of 70 week-old casp1^FL^ (black) and casp1^Cx3Cr1-KO^ (blue) *App^NL-G-F^* mice. Each symbol represents one mouse, n=5-6 (males, dark color; females, pale color). Data are represented as mean ± SEM. ns, not significant.

To infer plaque-associated axonal damage, we quantified dystrophic neural projections in the vicinity of the plaque by immune co-staining for N/25 and sAPP (**Figures 2M–P**). N/25 targets the Aβ_1-7_ epitope with high affinity and provides a global overview of amyloid deposition, while sAPP targets the amyloid precursor protein (APP). Early stages of Aβ-induced brain pathology involve axonal abnormalities, possibly associated with an atypical accumulation of APP and its cleavage products (43, 44). Microglial deletion of A20 on the *App^NL-G-F^* background did not induce different staining patterns or differences in the number of plaque-associated dystrophic neurites, suggesting that chronic microglial reactivity and neuroinflammation do not exacerbate the abnormal accumulation of full-length APP and Aβ at nerve terminals in *App^NL-G-F^* mice (**Figures 1M–P** and **Supplementary Figures 5I, J**).

Together, these results demonstrate that microglial deletion of A20 results in a condition of chronic hyperinflammation in the CNS without modulating Aβ pathology and axonal damage in the *App^NL-G-F^* mouse model of Aβ-induced brain pathology.

### Microglial caspase-1 deficiency does not suppress β-amyloid-induced pathology in *App^NL-G-F^* mice

To more directly assess the role of microglial inflammasome activation in Aβ-induced brain pathology, we next crossed mice carrying a floxed *caspase-1* allele (45) to *Cx3cr1* ^CreErt2^ mice (**Supplementary Figure 6A**). Control mice (*casp1^FL^*, expressing caspase-1) and mice with TAM-inducible caspase-1 deficiency in microglia (hereafter named *casp1^Cx3Cr1-KO^*) were subsequently crossed to *App^NL-G-F^* to examine the role of microglial caspase-1 activation in Aβ-induced pathology (**Supplementary Figure 6B**). Aβ-induced brain pathology was analyzed at the age of 20, 40 and 70 weeks with wild-type mice (*App^WT^*) included as negative controls (**Supplementary Figure 6C**). First, successful caspase-1 downregulation in microglia was confirmed at the protein level by Western blotting of FACS-sorted microglia *ex vivo* (**Supplementary Figure 6D**) and in primary microglia cultured *in vitro* (**Supplementary Figure 6E**).

As expected, amyloid plaques were readily observed in 20 week-old *App^NL-G-F^* mice (**Figures 2A, B** and **Supplementary Figures 7A–F**). Microglial caspase-1 deletion had no significant impact on amyloid plaque load (**Figure 2A** and **Supplementary Figures 7A–F**) or microglia numbers (**Figures 2C, D** and **Supplementary Figures 7G, H**) in *App^NL-G-F^* mice compared to that of caspase-1-sufficient *App^NL-G-F^* mice. Astrocyte numbers did not vary significantly across *APP^WT^* and *App^NL-G-F^* mice, irrespective of caspase-1 expression (**Figures 2E, F** and **Supplementary Figures 7I, J**).

No significant impact of microglial caspase-1 deletion could be observed in sAPP-N/25 immunoreactivity (**Figures 2G, H** and **Supplementary Figure 7K**), excluding an effect of caspase-1 activity on the extent of plaque-associated axonal damage. Finally, we analyzed potential differences in the number of plaque-associated microglia in *App^NL-G-F^* mice by co-staining for Iba1 and the fluorescent dye PFTAA (pentameric formyl thiophene acetic acid) that stains Aβ plaques. However, the number of plaque-associated microglia was unaltered upon caspase-1 deficiency (**Figure 2I**).

We speculated that the lack of effect of microglial caspase-1 deletion on Aβ-induced brain pathology might be explained by the amount of plaque load not having achieved a “critical level” in 20 week-old *App^NL-G-F^* mice. To test this hypothesis, we next assessed 40 week-old groups of casp1^FL^ *App^NL-G-F^* and casp1^Cx3Cr1-KO^ *App^NL-G-F^* mice for differences in Aβ-induced brain pathology. As *App^NL-G-F^* mice develop maximal plaque saturation by 7 months of age (35), this time point represents a late stage of Aβ-induced pathology. However, our analyses did not reveal significant differences in brain-wide amyloid plaque load when comparing casp1^FL^ *App^NL-G-F^* animals and casp1^Cx3Cr1-KO^ *App^NL-G-F^* animals (**Figures 2J, K** and **Supplementary Figures 8A–F**), nor differences in hippocampal microglia numbers (**Figures 2L, M** and **Supplementary Figures 8G, H**), or plaque and microglia phenotypes (**Figures 2J–M** and **Supplementary Figures 8A–H**). Also no differences were observed in hippocampal astrocyte numbers (**Figures 2N, O** and **Supplementary Figures 8I, J**), nor did the degree of plaque-associated axonal damage change significantly (**Figures 2P, Q** and **Supplementary Figure 8K**). In addition, we performed immunohistochemistry for Iba1, TMEM119 and Methoxy-04 (a fluorescent amyloid marker) to assess potential differences in the number of plaque-associated microglia in *App^NL-G-F^* cortical samples. However, no significant differences were observed between caspase-1-sufficient and caspase-1-deficient microglia (**Supplementary Figures 8L, M**). Microglial caspase-1 deletion did also not affect hippocampal or cortical Aβ_42_ loads in the casp1^FL^ *App^NL-G-F^* and casp1^Cx3Cr1-KO^ *App^NL-G-F^* groups (**Supplementary Figure 8N**). Finally, we assessed whole-brain plaque loads in very old (70 weeks) *App^NL-G-F^* mice, but again failed to identify significant differences between casp1^FL^ and casp1^Cx3Cr1-KO^ animals in relation to Aβ-plaque load (**Figure 2R**).

Taken together, these results demonstrate that microglial caspase 1 deletion has no significant impact on Aβ-induced brain pathology in the *App^NL-G-F^* mouse model of AD.

### Full-body inflammasome inhibition does not suppress ß-amyloid pathology in *App^NL-G-F^* mice

**Figure 3:**
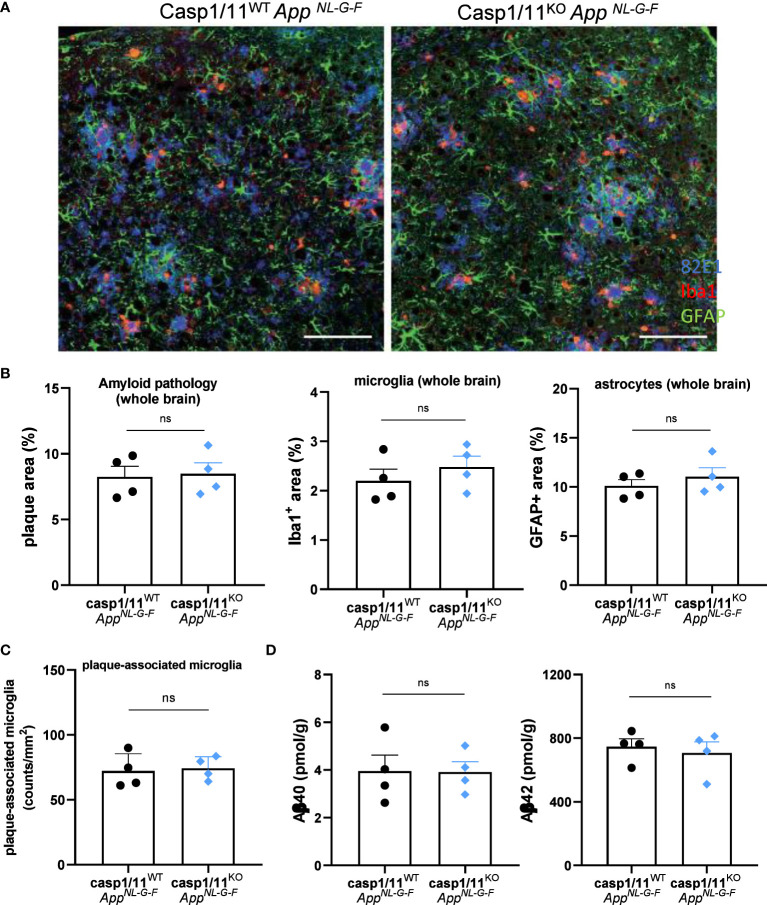
Full-body casp1/11 deficiency does not suppress AD pathology in 12 month-old *App^NL-G-F^* mice. **(A)** Amyloid deposition and neuroinflammation was detected by triple staining of 12-month-old casp1/11^WT^ and casp1/11^KO^ *App^NL-G-F^* mice using 82E1 (blue), anti-Iba1 antibody (red) and anti-GFAP antibody (green) as markers of Aβ plaque, astrocytosis and microgliosis, respectively. Scale bars represent 100 µm. **(B)** Quantification of 82E1+ amyloid plaque load, Iba1+ microgliosis, and GFAP+ astrocytosis over whole brains of casp1/11^WT^ and casp1/11^KO^ *App^NL-G-F^* mice (all females). Data are represented as mean ± SEM. **(C)** Quantification of the number of plaque-associated microglia in casp1/11^WT^ and casp1/11^KO^ *App^NL-G-F^* mice. Each symbol represents one mouse. Data are represented as mean ± SEM. **(D)** Biochemical quantification of Aβ_40_ and Aβ_42_ in the GuHCl fractions of cortical tissue from 12-month-old mouse brains from casp1/11^WT^ and casp1/11^KO^ *App^NL-G-F^* mice, quantified by sandwich ELISA. Each symbol is one mouse (all females), n=4 per group. Data represent mean ± SEM. ns, not significant.

Based on our unexpected findings with inducible microglia-specific caspase-1 deficient mice, we next evaluated whether constitutive full body caspase-1 deletion may display a more prominent impact on Aβ-induced brain pathology in the *App^NL-G-F^* model. To address potential roles of both canonical and non-canonical inflammasome pathways, we bred full-body caspase-1 knockout mice (46, 47) that are also deficient in caspase-11 (47) (casp1/11^KO^) to *App^NL-G-F^* mice, and assessed Aβ load and neuroinflammation in 12-month-old mice. Similar to our results with microglia-specific caspase-1-deficient mice, constitutive full body deletion of the central inflammasome effector proteases (Casp1/11^KO^ *App^NL-G-F^* mice) did not result in significant differences in Aβ plaque load, microgliosis, astrogliosis or in the number of plaque-associated microglia, compared to caspase-1/11-sufficient *App^NL-G-F^* mice (**Figures 3A–C**). Consistent herewith, no differences were observed in cortical Aβ_40_ and Aβ_42_ load between the two groups (**Figure 3D**).

### Microglial caspase-1 deletion and full-body Nlrp3 deletion fail to suppress ß-amyloid pathology in *APP/PS1* mice

**Figure 4:**
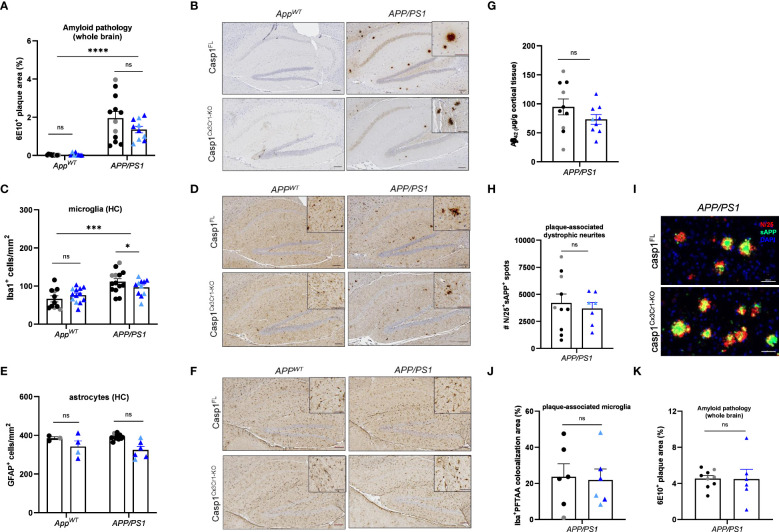
Microglial casp1 deficiency does not suppress AD pathology in *APP/PS1* mice. **(A)** Quantification of 6E10^+^ amyloid plaque pathology over whole brains of 20 week-old casp1^FL^ (black) and casp1^Cx3Cr1-KO^ (blue) *App^WT^* and *APP/PS1*. Each symbol represents one mouse, n=7-10 (*App^WT^*), n=10-12 (*APP/PS1*) (males, dark color; females, pale color). Data are represented as mean ± SEM. Significant differences are determined using two-way ANOVA (****p < 0.0001). **(B)** Immunohistochemistry for 6E10^+^ amyloid plaques in hippocampus of 20 week-old casp1^FL^ and casp1^Cx3Cr1-KO^ *App^WT^* and *APP/PS1* mice. Scale bars: 100 µm (inset: 50 µm). Representative images are displayed. **(C)** Quantification of the number of hippocampal Iba1^+^ microglia in of 20 week-old casp1^FL^ and casp1^Cx3Cr1-KO^ *App^WT^* and *APP/PS1*. Each symbol represents one mouse, n=7-13 (*App^WT^*), n=11-14 (*APP/PS1*). Data are represented as mean ± SEM. Significant differences are determined through two-way ANOVA (*p < 0.05; ***p < 0.001). **(D)** Immunohistochemistry for Iba-1^+^ microglia in the hippocampus of 20 week-old casp1^FL^ and casp1^Cx3Cr1-KO^ *App^WT^* and *APP/PS1*. Scale bars: 100 µm (inset: 50 µm). Representative images are displayed. **(E)** Quantification of the number of hippocampal GFAP^+^ astrocytes in of 20 week-old casp1^FL^ and casp1^Cx3Cr1-KO^ *App^WT^* and *APP/PS1*. Each symbol represents one mouse, n=3-5 per group (*App^WT^*), n=7-11 (*APP/PS1*). Data are represented as mean ± SEM. **(F)** Immunohistochemistry for GFAP^+^ astrocytes in the hippocampus of 20 week-old casp1^FL^ and casp1^Cx3Cr1-KO^ *App^WT^* and *APP/PS1 mice*. Scale bars: 100 µm (inset: 50 µm). Representative images are displayed. **(G)** Mean GuHCl-extractable Aβ_42_ levels in cortical brain tissue of 20 week-old casp1^FL^ and casp1^Cx3Cr1-KO^ *APP/PS1* mice. Each symbol represents one mouse, n=9-10 per group. Data are represented as mean ± SEM. **(H)** Quantification of the number of plaque-associated dystrophic neurites (N25^+^ sAPP^+^) in of 20 week-old casp1^FL^ and casp1^Cx3Cr1-KO^ *APP/PS1* mice. Each symbol represents one mouse, n=7-10 per group. Data are represented as mean ± SEM. **(I)** Immunohistochemistry for N25^+^ sAPP^+^ plaque-associated dystrophic neurites in the brain parenchyma of 20 week-old casp1^FL^ and casp1^Cx3Cr1-KO^ *APP/PS1* mice. Scale bars: 50 µm. Representative images are displayed. **(J)** Quantification of the number of plaque-associated microglia, measured as percentage Iba^+^ PFTAA colocalization area, in 20 week-old casp1^FL^ and casp1^Cx3Cr1-KO^ *APP/PS1* mice. Each symbol represents one mouse, n=6 per group. Data are represented as mean ± SEM. **(K)** Quantification of 6E10^+^ amyloid plaque pathology over whole brains of 70 week-old casp1^FL^ (black) and casp1^Cx3Cr1-KO^ (blue) *APP/PS1* mice. Each symbol represents one mouse, n=6-9 (*APP/PS1*) (males, dark color; females, pale color). Data are represented as mean ± SEM. ns, not significant.

Our results suggest that inflammasome activation is dispensable for Aβ-induced brain pathology in *App^NL-G-F^* mice with inducible microglia-selective caspase-1 deletion, and we extended these findings to *App^NL-G-F^* mice with a constitutive systemic deletion of the central inflammasome effector proteases caspases-1 and -11. We reasoned that defective inflammasome activation in the *App^NL-G-F^* model may have failed to recapitulate the decreased gliosis and plaque burden outcomes reported in *APP/PS1* mice with full-body deletion of caspase-1 or Nlrp3 (33) because *APP/PS1* mice overexpress APP, which is a non-physiological yet characteristic feature of that model (36). To empirically test this hypothesis, we next bred *casp1^Cx3Cr1-KO^* mice to *APP/PS1* mice and examined the role of microglial caspase-1 deletion in the Aβ-induced pathology of *APP/PS1* mutant mice. Unexpectedly, microglial caspase-1 deletion failed to significantly modulate Aβ plaque load in 20 week-old *APP/PS1* mice (**Figures 4A, B**). A significant, but minor effect was seen on microgliosis upon caspase-1-deficiency (**Figures 4C, D**). However, astrogliosis, plaque-associated axonal damage and the number of plaque-associated microglia were not significantly affected (**Figures 4E–J**). Also in aged (70 weeks old) *APP/PS1* mice, no significant difference in Aβ pathology was observed between casp1^FL^ *APP/PS1* and casp1^Cx3Cr1-KO^ *APP/PS1* mice (**Figure 4K**). Thus, similar to our previous observations in *App^NL-G-F^* mice (**Figures 1**–**3**), these results demonstrate that Aβ-induced brain pathology develops largely independently of microglial inflammasome activation in the *APP/PS1* mouse model.

**Figure 5:**
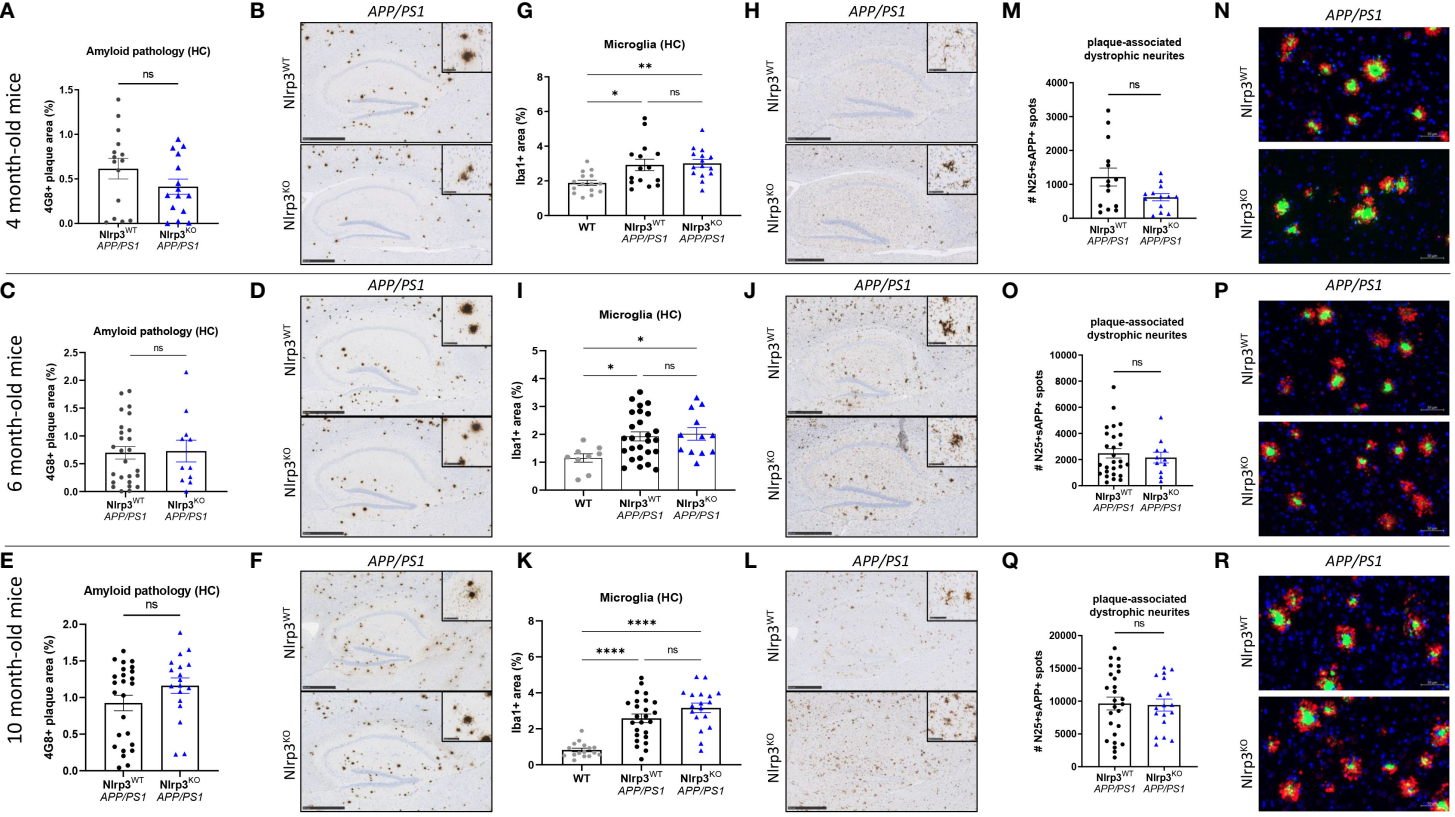
Full-body Nlrp3 deficiency does not suppress AD pathology in *APP/PS1* mice. **(A)** Quantification of 4G8^+^ amyloid plaque load in the hippocampus of 4-months old Nlrp3^WT^ and Nlrp3^KO^ *APP/PS1* mice. Each symbol represents one mouse. Data are represented as mean ± SEM. **(B)** Immunohistochemistry for 4G8^+^ amyloid plaque load in hippocampus of 4 month-old Nlrp3^WT^ and Nlrp3^KO^ *APP/PS1* mice. Scale bars: 500 µm (inset: 50µm). Representative images are displayed. **(C)** Quantification of 4G8^+^ amyloid plaque load in the hippocampus of 6-months old Nlrp3^WT^ and Nlrp3^KO^ *APP/PS1* mice. Each symbol represents one mouse. Data are represented as mean ± SEM. **(D)** Immunohistochemistry for 4G8^+^ amyloid plaque load in hippocampus of 6 month-old Nlrp3^WT^ and Nlrp3^KO^ *APP/PS1* mice. Scale bars: 500 µm (inset: 50µm). Representative images are displayed. **(E)** Quantification of 4G8^+^ amyloid plaque load in the hippocampus of 10-months old Nlrp3^WT^ and Nlrp3^KO^ *APP/PS1* mice. Each symbol represents one mouse. Data are represented as mean ± SEM. **(F)** Immunohistochemistry for 4G8^+^ amyloid plaque load in hippocampus of 10 month-old Nlrp3^WT^ and Nlrp3^KO^ *APP/PS1* mice. Scale bars: 500 µm (inset: 50µm). Representative images are displayed. **(G)** Quantification of the number of hippocampal Iba1+ microglia in 4 month-old Nlrp3^WT^ and Nlrp3^KO^ *APP/PS1* mice. Each symbol represents one mouse, n=14-23 per group. Data are represented as mean ± SEM. Significant differences are determined with One-way-ANOVA using Sidak’s multiple comparisons test (*p < 0.05; **p < 0.01). **(H)** Immunohistochemistry for Iba-1+ microglia in the hippocampus of 4 month-old Nlrp3^WT^ and Nlrp3^KO^ *APP/PS1* mice. Scale bars: 500 µm (inset: 50µm). Representative images are displayed. **(I)** Quantification of the number of hippocampal Iba1+ microglia in 6 month-old Nlrp3^WT^ and Nlrp3^KO^ *APP/PS1* mice. Each symbol represents one mouse, n=14-23 per group. Data are represented as mean ± SEM. Significant differences are determined with One-way-ANOVA using Sidak’s multiple comparisons test (*p < 0.05). **(J)** Immunohistochemistry for Iba-1+ microglia in the hippocampus of 6 month-old Nlrp3^WT^ and Nlrp3^KO^ *APP/PS1* mice. Scale bars: 500 µm (inset: 50µm). Representative images are displayed. **(K)** Quantification of the number of hippocampal Iba1+ microglia in 10 month-old Nlrp3^WT^ and Nlrp3^KO^ *APP/PS1* mice. Each symbol represents one mouse, n=14-23 per group. Data are represented as mean ± SEM. Significant differences are determined with One-way-ANOVA using Sidak’s multiple comparisons test (****p < 0.0001). **(L)** Immunohistochemistry for Iba-1+ microglia in the hippocampus of 10 month-old Nlrp3^WT^ and Nlrp3^KO^ *APP/PS1* mice. Scale bars: 500 µm (inset: 50µm). Representative images are displayed. **(M)** Quantification of the number of plaque-associated dystrophic neurites (N25+ sAPP+) in the brain of 4-months old Nlrp3^WT^ and Nlrp3^KO^ *APP/PS1* mice. Each symbol represents one mouse, n=14-23 per group. Data are represented as mean ± SEM. **(N)** Immunofluorescence staining for N/25 and sAPP in 4 months-old Nlrp3^WT^ and Nlrp3^KO^ *APP/PS1* mice. Scale bars: 50µm. Representative images are displayed. **(O)** Quantification of the number of plaque-associated dystrophic neurites (N25+ sAPP+) in the brain of 6-months old Nlrp3^WT^ and Nlrp3^KO^ *APP/PS1* mice. Each symbol represents one mouse, n=14-23 per group. Data are represented as mean ± SEM. **(P)** Immunofluorescence staining for N/25 and sAPP in 6 months-old Nlrp3^WT^ and Nlrp3^KO^ *APP/PS1* mice. Scale bars: 50µm. Representative images are displayed. **(Q)** Quantification of the number of plaque-associated dystrophic neurites (N25+ sAPP+) in the brain of 10-months old Nlrp3^WT^ and Nlrp3^KO^ *APP/PS1* mice. Each symbol represents one mouse, n=14-23 per group. Data are represented as mean ± SEM. **(R)** Immunofluorescence staining for N/25 and sAPP in 10 months-old Nlrp3^WT^ and Nlrp3^KO^ *APP/PS1* mice. Scale bars: 50µm. Representative images are displayed. ns, not significant.

To further expand our observations to *APP/PS1* mice with a systemic defect in NLRP3 inflammasome signaling, we finally evaluated the impact of constitutive full body Nlrp3 deletion (Nlrp3^KO^) on β-amyloid pathology by breeding Nlrp3 KO mice to *APP/PS1* mice. When assessing amyloid plaque deposition and neuroinflammation in 4, 6 and 10 month-old mutant mice, we found no significant differences in hippocampal or cortical Aβ plaque loads when comparing Nlrp3-sufficient and Nlrp3-deficient *APP/PS1* mice (**Figures 5A–F** and **Supplementary Figures 9A–F**). Microgliosis was observed from early age onwards in *APP/PS1* mice, but Nlrp3 deletion did not modulate microglia numbers in the hippocampus or cortex (**Figures 5G–L** and **Supplementary Figures 9G–L**). At young age (4 months), we observed a trend toward a reduced number of dystrophic neurites in Nlrp3^KO^ *APP/PS1* mice, although it did not reach statistical significance. Furthermore, no differences between the two genotypes were observed at older age in this regard (**Figures 5M–R**). We also quantified neuronal counts by immunohistochemistry using the NeuN antibody, but again observed no clear differences between Nlrp3-deficient and Nlrp3-sufficient *APP/PS1* mice (**Supplementary Figures 10A–D**). Finally, Aβ40 and Aβ42 levels were also similar in the two genotypes (**Supplementary Figures 10E, F**). Contrasting a previous report (33), these results show that full-body Nlrp3 deletion has little measurable impact on Aβ-induced neuropathology in *APP/PS1* mice.

### Nlrp3 signaling does not shape microglial activation during homeostasis or upon amyloid pathology in *APP/PS1* mice

Single-cell RNA sequencing (scRNA-seq) approaches have revealed the nature of microglial responses to AD-associated amyloid pathology (48–50). Previous studies have identified a disease-associated microglia (DAM) signature upon amyloid pathology, with DAMs exhibiting a downregulation of homeostatic signature genes and an induction of genes associated with phagocytosis and lipid metabolism (48–50).

**Figure 6:**
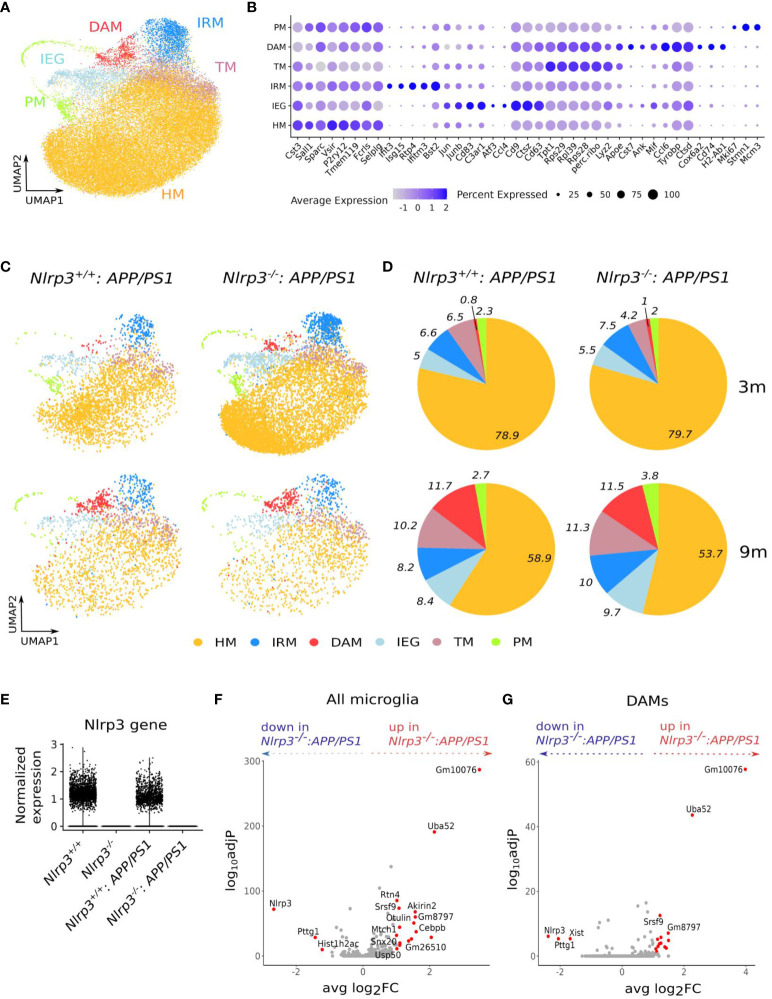
Nlrp3 signaling does not shape microglial activation during homeostasis or upon amyloid pathology in *APP/PS1* mice. **(A)** UMAP plot of 40547 microglia cells from whole brain tissue of *Nlrp3* ^+/+^ mice (3, 6 and 9 months old), *Nlrp3* ^-/-^ mice (3, 6 and 9 months old), *Nlrp3* ^+/+^ *APP/PS1* (3 and 9 months old) and *Nlrp3* ^-/-^ *APP/PS1* (3 and 9 months old). **(B)** Dot plot, showing the expression of key marker genes per cluster in the dataset from (a) The size of the dot represents the percentage of cells within a cluster that express the gene, while the color encodes the mean scaled gene expression level per cluster. **(C)** UMAP plot of microglia from *Nlrp3* ^+/+^ *APP/PS1* and *Nlrp3* ^-/-^ *APP/PS1* mice, split by genotype and age. **(D)** Pie charts visualizing the percentage of cells per cluster in each genotype and age group from (c) **E**. Violin plot, comparing the expression level of the Nlrp3 gene in the microglia from the four distinct genotypes (all age groups). **(F)** Volcano plot, showing differentially expressed genes between microglia (all clusters combined) from *Nlrp3* ^-/-^ *APP/PS1 vs Nlrp3* ^+/+^ *APP/PS1* mice (9 months old). In red are shown the genes with adj P<0.05 and abs(log2FC)>1. **(G)** Volcano plot, showing differentially expressed genes between DAMs from *Nlrp3* ^-/-^ *APP/PS1 vs Nlrp3* ^+/+^ *APP/PS1* mice (9 months old). In red are shown the genes with adj P<0.05 and abs(log2FC)>1. HM, homeostatic microglia; IRM, Interferon response microglia; TM, transitory microglia; DAM, disease-associated microglia; IEG, immediate early genes response microglia; PM, proliferating microglia.

To investigate whether microglial activation in the context of amyloid pathology is shaped by Nlrp3 signaling, we performed scRNA-seq on CD45^+^ brain immune cells isolated from 4 groups of mice: wild-type (WT), Nlrp3^KO^, *APP/PS1* and Nlrp3^KO^ *APP/PS1* mice. Furthermore, mice were profiled at the age of 3, 6 and 9 months for WT and Nlrp3^KO^ mice, and at the age of 3 and 9 months for the *APP/PS1* and the Nlrp3^KO^ *APP/PS1* groups. In each of the 10 individual scRNA-seq datasets, microglia were identified based on known signature genes (50) and were subsequently pooled in a single dataset (**Figure 6A**). In accordance with previous studies (48–50), we were able to identify microglial cell states that correspond to homeostatic microglia (HM), DAMs, microglia transitioning toward DAMs (TM), microglia with an IFN-signature (interferon-response microglia or IRM), proliferating microglia (PM) and a cluster exhibiting an immediate-early gene (IEG) signature, which is a known response to tissue-dissociation (50, 51) (**Figures 6A, B**). As reported previously (50), DAMs were enriched in *APP/PS1* mice as compared to their non-diseased WT littermates (**Figures 6C, D** and **Supplementary Figures 11A, B**). Remarkably, the relative proportion of the different microglial cell states, including DAMs, was comparable between Nlrp3^WT^ and Nlrp3^KO^ mice, both in the non-diseased (**Supplementary Figures 11A, B**) and *APP/PS1* backgrounds (**Figures 6C, D**). Furthermore, in healthy non-diseased brains, microglia from Nlrp3 sufficient and Nlrp3 deficient mice showed only a few differentially expressed genes, across all age groups (**Supplementary Figures 11C–E**). The only significantly upregulated gene in Nlrp3^KO^ microglia was the predicted pseudogene *Gm8797*. The strongest downregulated gene was *Nlrp3*, which was completely absent in the Nlrp3^KO^ groups (**Figure 6E**), confirming its complete deletion. Similarly, only a limited number of genes were differentially expressed between Nlrp3 WT and KO microglia within the *APP/PS1* background both at 3 and 9 months of age (**Figure 6F**; **Supplementary Figure 12A**), and the top differentially expressed genes included multiple pseudogenes, predicted genes and ribosomal genes (e.g. *Gm10076, Gm26510, Rps29, Rps28*). The same was observed for DAMs from Nlrp3^WT^ and Nlrp3^KO^ *APP/PS1* mice (**Figure 6G**). We also did not identify a robust NF-κB (*Rela, Nfkbia, Relb, Ccl2, Ccl5, Il6, Tnfaip3, Tnf, Il1b*) or inflammasome signature (*Nlrp3, Nlrp1b, Pycard, Casp1, Casp4, Gsdmd, Il1b, Il18*) in DAMs or other microglial clusters in *APP/PS1* mice, and these genes were not altered upon *Nlrp3* deficiency (**Supplementary Figure 12B**). Overall, these single cell transcriptomics data suggest that inflammasome activation is not a central regulator of microglial activation in the healthy brain or upon amyloid pathology in the *APP/PS1* model.

## Discussion

Stimulation of microglia with fibrillar Aβ leads to a hyperinflammatory response that is characterized by the upregulation and activation of the Nlrp3 inflammasome (32). This activation subsequently triggers the production and secretion of pro-inflammatory cytokines IL-1β and IL-18 (32). Activation of the microglial Nlrp3 inflammasome has been proposed as a key contributor to the progression of AD pathology in the *APP/PS1* and *CRND8-APP* models of Aβ-induced neuropathy (33, 52). *APP/PS1* mice are double transgenic mice that express a mutant amyloid precursor protein (Mo/HuAPP695swe or APP-K670N/M671L) together with a mutant human presenilin-1 (exon 9-deleted PS1 or PS1-L166P) (34, 53), while CRND8 mice transgenically express a mutant *APP* incorporating the double Swedish (K670N/M671L) and Indiana (V717F) mutations (54). These mutations are all associated with early-onset Alzheimer’s disease.

To further investigate the role of the Nlrp3 inflammasome in a different model of Aβ-induced neurodegeneration, we first conducted experiments in *App^NL-G-F^* mice with a microglia-selective deficiency in A20, an anti-inflammatory protein that suppresses Nlrp3 inflammasome activation (**Supplementary Figure 1A**). We also examined the effect of microglial deficiency in caspase-1, the central effector protease of the Nlrp3 inflammasome (**Supplementary Figure 1B**). Notably, our approach utilizing the Cx3Cr1^CreERT2^ deletion strategy allows for selective targeting of long-lived brain macrophages, while excluding chronic gene deletion in peripheral myeloid cells (41). Our findings demonstrated that microglial inflammasome activation plays a negligible role in Aβ-induced neuroinflammation and brain pathology in *App^NL-G-F^* mice. This conclusion is further supported by our investigation into the potential consequences of constitutive full-body deletion of caspases-1 and -11 in *App^NL-G-F^* mice (**Supplementary Figure 1B**), which provided evidence that constitutively impaired inflammasome signaling in all cell types of the body has minimal impact on the development of Aβ-induced brain pathology in diseased *App^NL-G-F^* mice.

To corroborate our findings and further explore a potential role of microglial inflammasome activation in other mouse models of Aβ-induced neuropathology, we extended our studies to the widely used *APP/PS1* mouse model of AD. Unexpectedly, our experiments involving the selective inactivation of caspase-1 in microglia of *APP/PS1* mice (**Supplementary Figure 1B**) yielded similar results to what was observed for *App^NL-G-F^* mice. Moreover, our results revealed that full body constitutive deletion of Nlrp3 in *APP/PS1* mice (**Supplementary Figure 1C**) did not mitigate the development of Aβ-induced neuropathology. These findings further strengthened our conclusion that inflammasome activation has a negligible effect on Aβ-induced neuropathology, and provide a strong rationale for questioning a generalizable critical role of inflammasome activation in mouse models of ß-amyloid-induced neuroinflammation and AD-associated amyloid pathology.

Finally, by conducting a comparative analysis of microglia using scRNASeq in Nlrp3 knockout and wild-type *APP/PS1* mice, we revealed that the relative proportions of microglial cell states, including disease-associated microglia (DAMs), were similar between Nlrp3-sufficient and Nlrp3-deficient conditions. This intriguing finding suggests that the absence of Nlrp3 and subsequent inflammasome activation does not significantly impact the distribution of microglial cell states in response to Aβ-associated pathology.

The role of inflammasome activation in the pathogenesis of AD is an area of active research and ongoing debate. It is clear that Aβ fibrils activate the Nlrp3 inflammasome in microglia (32), and several reports have identified activated inflammasome effectors - in particular caspase-1 and IL-1ß - in the brains of AD patients and in animal models of AD-associated neuropathology (30, 31, 33, 52, 55–61). However, other published findings suggest a more complex involvement of IL-1ß secretion in AD pathology. Sustained transgenic overexpression of IL-1ß was shown to have a beneficial effect on amyloid burden without overt neurodegeneration in the APP/PS1 and APPswe/PS-1dE9 mouse models of Aβ-associated neuropathy (62, 63). IL-1 Receptor (IL-1R1) deficiency also did not appear to modulate Aβ loads in aging APP-transgenic (Tg2576) mice (64). Moreover, IL-1ß-induced neuroinflammation was shown to regulate Aβ and tau pathology in opposing ways in the triple transgenic mouse model of AD (65).

The work presented here suggests that the Nlrp3 inflammasome is not critically involved in shaping microglial activation states or in driving Aβ-induced neurodegeneration in preclinical models of Aβ-associated neuropathy. It is worth mentioning that our conclusions are based on observations in two different animal models of Aβ-associated neuropathy (APP/PS1 and *App^NL-G-F^* mice), and involved both microglia-selective as well as constitutive full body deletion of core inflammasome components (caspase-1 and Nlrp3). Although we can only guess the reasons underlying the discrepant results obtained in our study and those of a previous report (33), potential explanations may include variations in experimental and environmental factors (**Supplementary Table 1**). First, genetic differences in AD models may explain the discrepant results between our and previous studies. The APP/PS1 model used in this study is the widely used model developed by the Jucker lab (34), which is a relatively ‘aggressive’ Aβ model with a high Aβ42/40 ratio. In contrast, the Heneka study used a different APP/PS1 mouse strain that is characterized by a slower plaque growth (53). Moreover, the *App^NL-G-F^* mice used in our study do not rely on transgenic overexpression as in the APP/PS1 models, but instead carry coding sequence mutations in the endogenous *APP* gene that drive rapid Aβ aggregation, and thus may be less sensitive to microglia-dependent modulation of Aβ-induced neuropathology. Second, variations in both the mice’s gender and age during analysis could contribute to distinct observations. In our study, mice underwent analysis at both initial and advanced stages of disease progression, while the Heneka study reportedly conducted analysis at 16 weeks of age (**Supplementary Table 1**). Third, the composition of host microbiota linked to chow and other environmental factors in different animal facilities might contribute to differences in inflammasome responses. Indeed, the intestinal microbiota was shown to modulate immune functions and signaling to a wide variety of distant cells, including microglia (66, 67). It is worth mentioning in this regard that our studies were performed in three different animal facilities (VIB-Ghent University in Ghent, Belgium; Janssen Pharmaceutica in Beerse, Belgium; RIKEN Center for Brain Science, Saitama, Japan) with similar outcomes. Therefore, even though an animal facility-specific effect of differential microbiomes remains possible, additional examination in independent facility environments would be required to endorse putative effects of the intestinal microbiota.

Regardless, by demonstrating that the role of the Nlrp3 inflammasome in Aβ-induced neurodegeneration is not as prominent or universal as previously anticipated, our findings contribute to the ongoing debate and highlight the need for further investigation into the complex mechanisms underlying AD. Although our findings demonstrate that inflammasome activation does not play a significant role in mouse models of Aβ-induced neurodegeneration, these observations leave open the possibility that inflammasomes may be implicated in the response to other mechanisms contributing to AD pathology, including the aggregation of hyperphosphorylated tau and the formation of neurofibrillary tangles. Loss of Nlrp3 inflammasome function was recently shown to reduce tau hyperphosphorylation and aggregation, identifying an important role of microglia and Nlrp3 inflammasome activation in the pathogenesis of tauopathies (68). With the emergence of brain-penetrant Nlrp3 inhibitors, future investigations should focus on examining whether pharmacological targeting of the Nlrp3 inflammasome can effectively suppress the formation of neurofibrillary tangles in AD and other tauopathies. Such studies could provide valuable insights into the mechanisms underlying neurodegeneration and facilitate the development of targeted therapeutic strategies for tau-related diseases.

## Material and methods

### Animals

*App^NL-G-F^* (35), *APP/PS1* (34), caspase-1/11 (casp1/11) knockout (46) and Nlrp3 knockout (69) mice have been described. Casp1/11^KO^ mice were crossed with *App^NL-G-F^* mice to generate Casp1/11^KO^ *App^NL-G-F^* mice. Nlrp3^KO^ mice were crossed with *APP/PS1* mice to generate Nlrp3^KO^ *APP/PS1* mice. Conditional A20 (40) and Casp1 (45) knockout mice were generated as previously described, and crossed with Cx3Cr1Ert2-Cre transgenic mice (41) to generate tamoxifen-inducible myeloid-specific A20 and caspase-1 knockout mice, and further crossed with *App^NL-G-F^* transgenic mice. A20^FL^ and Casp1^FL^ littermate mice, expressing the floxed allele, are used as controls in all experiments. Casp1^FL^ and Casp1^Cx3Cr1-KO^ mice were also crossed with *APP/PS1* transgenic mice. At 4-6 weeks of age, mice were subcutaneously injected with tamoxifen (20 mg/ml, Sigma-Aldrich T5648) dissolved in corn oil (Sigma-Aldrich, C8267) twice, 48h apart, to activate Cre recombinase. All experiments were performed on mice backcrossed into the C57BL/6 genetic background for at least 8 generations. Mice were housed in individually ventilated cages in a specific pathogen-free facility at VIB-Ghent, Belgium, at RIKEN and Nagoya City University, Japan (for the Casp1/11-*App^NL-G-F^* line), or at Janssen Pharmaceutica Beerse, Belgium (for the Nlrp3^KO^ *APP/PS1* mice). All experiments were conducted according to institutional, national, and European animal regulations or in accordance with the guidelines of the RIKEN Center for Brain Science and Nagoya City University. Animal protocols were approved by the ethics committee of Ghent University, by the ethics committee of RIKEN Center for Brain Science and Nagoya City University, or by the ethics committee of Janssen Pharmaceutica.

### Histology, brightfield microscopy and image analysis

Mice were transcardially perfused with PBS (Gibco). Brain tissue was dissected and left overnight at 4˚C in formaldehyde-based fixative before being embedded in paraffin blocks. Alternatively, for biochemical analyses, hippocampi were dissected out and snap-frozen in liquid N_2_ until further processing. Sagittal sections of 5 µm were rehydrated and incubated in antigen retrieval buffer (Dako). Analyses were performed over whole brain, hippocampus (bounded by CA1 and dentate gyrus), and cortex regions (“cortex” refers to areas in the mouse brain labeled by the Mouse Brain Atlas as visual, posterior parietal association areas, somatosensory, somatomotor, and orbital cortices, fiber tracts excluded) as clarified in individual graphs. For 6E10 staining, antigen retrieval was performed by incubating sections with 33% formic acid for 20 minutes. Endogenous peroxidase activity was blocked by treating tissue with 3% H_2_O_2_ for 8-10 minutes. Non-specific binding was blocked by treating slides with blocking buffer (0.5% fish-skin gelatin + 2% BSA in PBS + 5% goat serum) for minimum 1h. The primary antibodies Iba1 (1:1000; Wako Chemicals), GFAP (1:5000; Dako), 6E10 (1:500; Biolegend), and 82E1 (1:1000, IBL America) were incubated overnight at 4˚C. Tyramide signal amplification (PerkinElmer Life Sciences) was used, as previously described (70). Brightfield images were obtained using Axio Scan.Z1 (Zeiss, Germany). Iba1^+^ and GFAP^+^ cell bodies were manually counted in 7-12 representative images per experimental group using Zen (blue edition) (Zeiss). Image visualization parameters were standardized, and hippocampal glia numbers were normalized to hippocampal area (manually demarcated). Quantification of GFAP^+^ area and 6E10^+^ plaque area over the whole brain was quantified using QuPath software (71). Graphs represent % of total brain area positive for GFAP^+^ or 6E10^+^ staining.

### Immunofluorescence microscopy and image analysis

For fluorescent immunohistochemistry of plaque-associated dystrophic neurites, rehydrated sections were incubated in 70% formic acid for 10 minutes. Endogenous peroxidase was blocked with H_2_O_2_ treatment, and slides were incubated overnight with the primary antibodies JRD/sAPP/32 (0.2 µg/ml, kind gift J&J, Beerse-Belgium) and JRF/AβN/25 (2 µg/ml, kind gift J&J, Beerse-Belgium) in antibody diluent (DAKO). After washing, slides were incubated for one hour with secondary antibodies anti-mouse IgG2a Alexa647 (1:500, Life Technologies) and anti-mouse IgG1 Alexa488 (1:500, Life Technologies). For fluorescent immunohistochemistry of plaque-associated microglia, sections were incubated with Iba1 antibody (Wako Chemicals, Richmond, VA, USA) overnight, followed by incubation with the fluorescent dye PFTAA (pentameric formyl thiophene acetic acid, J&J, Beerse-Belgium) for one hour. Endogenous peroxidase activity was blocked with 3% hydrogen peroxide (DAKO, Glostrup, Denmark). Alternatively, 3 µm thick formaldehyde-fixed paraffin-embedded tissue sections were deparaffinized and cooked in EnVision FLEX Target Retrieval Solution High pH (DAKO) for 40 minutes, and blocked in 1% BSA, 1% Triton-X-100 in PBS. The slides were then incubated with antibodies against Iba1 (Synaptic Systems, 1:500) and TMEM119 (Synaptic Systems, 1:200) in 1% BSA, 1% Triton-X-100 in PBS at 4°C overnight. Sections were then washed and incubated for 2 hours with secondary antibodies Donkey Anti-Chicken Alexa Fluor 488 (Jackson Immuno, 1:300) and Donkey Anti-Rabbit Alexa Flour 568 (ThermoFisher, 1:500). Slides were then incubated with Methoxy-X04 (Tocris, 1:3000) to visualize plaques. Imaging was performed with a BZ-X800 fluorescent microscope (Keyence) and a 20x objective. At least 100 plaque-associated and 100 non-plaque-associated cortical Iba1 positive cells per animal were evaluated for TMEM119 expression by manual inspection. Quantification of sAPP-labeled dystrophic neurites near N/25-positive plaque deposits was performed using Columbus image analysis software. Thresholds and plaque area were kept constant for all samples. Using QuPath software (71), N25/sAPP and Iba1/PFTAA images were sub-sampled into one training image and a pixel classifier that has been trained using Random Tree Forest by manually annotating colocalization and non-colocalization areas. For each slide, tissue area was detected by applying a gaussian blur of 2 pixels followed by a threshold on the DAPI channel, and pixels were classified using the pixel classifier. Colocalization detection was measured by tissue area.

### Quantitative real-time PCR on mouse hippocampal tissue

Total RNA was isolated using TRIzol reagent (Invitrogen) and Aurum Total RNA Isolation Mini Kit (Biorad), following manufacturer’s instructions for processing animal tissue. cDNA was synthesized using Bioline cDNA synthesis kit (Bioline). 10 ng cDNA was used for quantitative PCR in a total volume of 10 uL with LightCycler 480 SYBR Green I Master Mix (Roche) with appropriate primers, on a LightCycler 480 (Roche). All reactions were performed in triplicate. Mouse-specific primers used are as follows: *Hprt* forward 5’-AGTGTTGGATACAGGCCAGAC-3’ reverse 5’-CGTGATTCAAATCCCTGAAGT-3’, *Gapdh* forward 5’-TGAAGCAGGCATCTGAGGG-3’ reverse 5’-CGAAGGTGGAAGAGTGGGAG-3’, *CCL5* forward 5’-CGTCAAGGAGTATTTCTACAC-3’ reverse 5’-GGTCAGAATCAAGAAACCCT -3’, *CCL2* forward 5’-TTAAAAACCTGGATCGGAACCAA-3’ reverse 5’-GCATTAGCTTCAGATTTACGGGT-3’, *proIL-1β* forward 5’-TGGGCCTCAAAG GAA AGA-3’ reverse 5’-GGTGCTGATGTACCA GTT-3’, *proIL-18* forward 5’-CAGGCCTGACATCTTCTGCAA-3’ reverse 5’-TCTGACATGGCAGCCATTGT-3’, *IL-6* forward 5’-GAGGATACCACTCCCAACAGACC-3’ reverse 5’-AAGTGCATCATCGTTGTTCATACA-3’, *TNF* forward 5’-ACCCTGGTATGAGCCCATATAC-3’ reverse 5’- ACACCCATTCCCTTCACAGAG-3’.

### Sandwich Ab40 and Aß_42_ Meso Scale Discovery (MSD) assay and Aß_40-42_ ELISA

Brain fractions were homogenized in ice-cold 5 M Guanidine-HCl and 50 mM Tris/HCl extraction buffer. After homogenization, samples were placed in an end-over-end rotation wheel (3 h, RT). Brain homogenates were stored at -80°C. Synthetic human Ab1-40 and Aß1-42 peptides in DMSO (AnaSpec, 100 µg/mL) were diluted in dilution buffer (0.5 M GuHCl + 5 mM Tris-HCl - pH 8.0; 10-fold dilution of extraction buffer in 0.1% casein in PBS and applied as standards). GuHCl extracts were diluted 10-fold in ice-cold 0.1% casein in PBS and centrifuged (14000 rpm, 4°C, 20 min). Supernatant was further diluted in dilution buffer. 96-well plates (Meso Scale Discovery) were coated with monoclonal capture antibodies overnight (JRF/Ab40/28 or JRF/Aß42/26, 1.5 µg/mL in PBS, 4°C). The next day, plates were washed and blocked with 0.1% casein in PBS (2 h, RT). After a washing step, standards and samples were incubated with 3D6-SULFO antibody overnight (4°C). After washing, 2x MSD Read Buffer was added and plates were read on MSD Sector Imager 6000. Aß_40_ and Aß_42_ levels in brain extracts were also quantified using an Aß ELISA kit (Wako) according to the manufacturer’s instructions.

### Western blotting

Equally seeded cell cultures were lysed in lysis buffer (20 mM Tris HCl (pH 7.4), 200 mM NaCl, 1% Nonidet P-40) and were denatured in 4x Laemmli buffer. Proteins were separated by Sodium Dodecyl Sulfate Polyacrylamide Gel Electrophoresis (SDS-PAGE), transferred to a nitrocellulose membrane followed by incubation overnight at 4°C with primary antibody against A20 (1:1000, SC- 166692, Santa Cruz Biotechnology), caspase-1 p20 (1:1000, SC-22165, Santa Cruz) and HRP-conjugated secondary goat anti-rabbit (1:2500, DAKO) in Tris-buffered Saline supplemented with 0.05% Tween-20 and 5% nonfat dry milk. Detection was performed with chemiluminescence (Western Lightning Plus-ECL, PerkinElmer) using an Amersham Imager 600 (GE Healthcare). After imaging, blots were incubated with directly labeled primary antibody β-actin-HRP (1:10000, Santa Cruz) for 15 minutes as loading control.

### Microglia isolation for *in vitro* experiments

0-3 day-old pups were used to isolate primary microglia. Brains were stripped of olfactory bulbs, cerebellum, midbrain, and meninges and stored in ice-cold F12 Nutrient Mix Ham medium (Gibco Life Technologies), after which, the tissue was trypsinized and resuspended in DMEM medium supplemented with 10% FCS, 1% penicillin/streptomycin, glutamine, sodium pyruvate, and non-essential amino acids (NEAA). Cells were grown in tissue flasks (75cm^3^) pre-treated with poly-L-lysine for a minimum of 1h. Medium was refreshed approximately every other day until a confluent astrocyte layer appears (in 7-10 days). Microglia were then generated by changing the medium composition to 75% of DMEM medium + 25% L929 conditioned medium. After 4-6 days, microglia were isolated from this mixed glial culture by shaking the flasks at 100 rpm at 37°C for 1h and plated with 75% RPMI medium (supplemented same as DMEM) + 25% L929 conditioned medium. Cre-mediated deletion was induced by treating plated microglia with 1µM 4-hydroxytamoxifen (4-OH-TAM, Sigma) for 3 days.

### Microglia isolation from adult mice

Mice were perfused with PBS containing 5 IU/ml heparin. Isolated brains were stored on ice in HBSS with 45% glucose and HEPES and subsequently minced with a scalpel and dissociated in high-glucose DMEM containing collagenase A, FCS, and DNAse I (30 minutes, 37°C). Cells were centrifuged (10 min, 400*g*, 4˚C) and resuspended in 5 ml of 25% Percoll overlaid with 3 ml of ice-cold PBS. Microglial cells were obtained as a pellet after centrifugation (30 min, 800*g*, 4°C), and resuspended in FACS buffer (0.5% BSA, 2mM EDTA in PBS). Microglia were stained (30 min, on ice, in the dark) using anti-CD45-PE (1:800; clone 30-F11, eBioscience), anti-CD11b-APC-Cy7 (1:400; clone M1/70, BD Biosciences), and Fc receptor blocking antibody CD16/CD32 (1:400; clone 2.4G2, BD Biosciences). Microglia were sorted as CD45int CD11b+ cells with an Aria II (BD Biosciences) to >90% purity.

### Statistical analysis

Each n represents an independent biological sample. All data are represented as mean ± S.E.M. Statistical analysis was done using GraphPad Prism software version 8.0. and Genstat. Data were analyzed by applying statistical tests depending on the distribution of the data. Type of statistical analysis is mentioned for each experiment.

### Single-cell RNA sequencing and analysis

Nlrp3^-/-^ mice were crossed to *APP/PS1* transgenics to obtain the following four groups of littermates: Nlrp3^+/+^; Nlrp3^-/-^; Nlrp3^+/+^ *APP/PS1* ^Tg^; Nlrp3^-/-^ *APP/PS1* ^Tg^. Littermates were collected at 3, 6 (only for Nlrp3^+/+^ and Nlrp3^-/-^ mice) and 9 months of age. For each group, 3 mice (2 males and 1 female) were killed and used for brain isolation and single-cell processing. All processing and tissue-collection was performed as previously described (72), using actinomycin D to limit dissociation-induced gene expression (50). All steps were performed at 11°C. In brief, the brain was extracted and placed in ice-cold RPMI (Gibco), containing 30 μM Actinomycin D (ActD) (Sigma, No. A1410). The brains were split in half and each hemisphere was cut into small pieces and incubated with enzyme mix [30 U ml−1 DNAse I (Roche), 10 U ml−1 collagenase type I (Worthington) and 400 U ml−1 collagenase type IV (Worthington) diluted in 1× Hanks’ buffered salt solution (Gibco)], containing 15 μM ActD at 11°C for 40 min. Every 10 min the solution was cut and resuspended to ensure full dissociation of the tissue. Subsequently, the solution was resuspended, filtered twice over a 100 μm nylon filter, using RPMI with 3µM ActD and centrifuged. The pellet was resuspended in 5 ml 70% standard isotonic percoll (SIP, GE Healthcare) diluted in 1× Hanks’ buffered salt solution and gently overlaid with 5 ml of 37% SIP, followed by a 5 ml layer of 30% SIP, forming a three-layered density gradient (centrifuged at 800g, 4°C, 30 min without acceleration/braking). All gradient buffers contained 3µM ActD. The 70/37% interphase containing immune cells was collected, centrifuged and resuspended in fluorescent activated cell sorter (FACS) buffer (2 mM EDTA (Duchefa), 2% heat-inactivated fetal calf serum (Gibco) dissolved in 1× Hanks’ buffered salt solution), containing 3µM ActD. The cell suspensions of n=3 mice per genotype (Nlrp3^+/+^, Nlrp3^-/-^, Nlrp3^+/+^ *APP/PS1* ^Tg^, Nlrp3^-/-^ *APP/PS1* ^Tg^) were pooled, per age group, respectively. The dissociation process required 4 hours. Following single-cell isolation, cells were blocked with rat anti-mouse CD16/CD32 (clone 2.4G2) for 15 min on ice. Subsequently, the cells were stained with anti-CD45-APCCy7 (30-F11, BioLegend) for 20 min on ice and washed. All CD45+ immune cells were sorted in ME-medium [RMPI medium supplemented with 20% heat-inactivated fetal calf serum (Gibco), 300 μg ml-1 l-glutamine (Gibco), 100 units ml–1 penicillin and 100 μg ml–1 streptomycin (Gibco), 1 mM non-essential amino acids (Gibco), 1 mM sodium pyruvate (Gibco) and 0.05 mM 2-mercaptoethanol (Sigma)], containing 3µM ActD, using a BD FACS ARIA II, or FACS ARIA III, with a sorting nozzle of 85 µm. DAPI (Sigma) was used to exclude dead cells; cell viability before and after cell sorting exceeded 90%. Sorted cells were centrifuged at 4°C at 400g, then resuspended in PBS + 0.04% bovine serum albumin at room temperature to yield an estimated final concentration of 1,000 cells μl^–1^.

Cellular suspensions were loaded on a Chromium Chip B (10x Genomics, No.1000074) on a GemCode Single Cell Instrument (10x Genomics) to generate single-cell gel beads-in-emulsion (GEM). GEMs and scRNA-seq libraries were prepared using the GemCode Single Cell 3’ Gel Bead and Library Kit (v3 10xGenomics, No. 1000075) and the Chromium i7 Multiplex Kit (10x Genomics, No. 120262) according to the manufacturer’s instructions. Briefly, GEM reverse-transcription incubation was performed in a 96-deep-well reaction module at 53°C for 45 min, 85°C for 5 min and ending at 4°C. Next, GEMs were broken and complementary DNA (cDNA) was cleaned up with DynaBeads MyOne Silane Beads (10x Genomics, No. 2000048) and SPRIselect Reagent Kit (Beckman Coulter, No. B23318). Full-length, barcoded cDNA was PCR amplified with a 96-deep-well reaction module at 98°C for 3 min, eleven cycles at 98°C for 15 s, 63°C for 20 s and 72°C for 1 min, followed by one cycle at 72°C for 1 min and ending at 4°C. Following cleaning up with the SPRIselect Reagent Kit and enzymatic fragmentation, library construction to generate Illumina-ready sequencing libraries was performed by the addition of R1 (read 1 primer), P5, P7, i7 sample index and R2 (read 2 primer sequence) via end-repair, A-tailing, adapter ligation, post-ligation SPRIselect cleanup/size selection and sample index PCR. The cDNA content of pre-fragmentation and post-sample index PCR samples was analyzed using the 2100 BioAnalyzer (Agilent).

Sequencing libraries were loaded on an Illumina HiSeq4000 flow cell with sequencing settings following the recommendations of 10x Genomics (Read 1: 28 cycles, i7 Index: 8 cycles, i5 Index: 0 cycles, Read 2: 91 cycles, 2.73nM loading concentration). The Cell Ranger pipeline (10x Genomics) was used to perform sample demultiplexing and to generate FASTQ files for read 0, read 2 and the i7 sample index. Read 2, containing the cDNA, was mapped to the reference genome (mouse mm10) using STAR. Subsequent barcode processing, unique molecular identifiers filtering and single-cell 3’ gene counting was performed using the Cell Ranger suite and Seurat v.4.3.1. The total number of cells across all libraries was 47 633 cells. The average of the mean reads per cell across all libraries was 45 319.6 ± 22 166 SD, with an average sequencing saturation of 67.05% ± 14.21% SD, as calculated by Cell Ranger. Digital gene expression matrices were preprocessed and filtered using the Seurat and Scater (v.1.22.0) R packages. Outlier cells were identified based on three metrics (library size, number of expressed genes and mitochondrial proportion per cell); cells were tagged as outliers when they were more than three median absolute deviations distant from the median value of each metric across all cells. By means of the Seurat Merge function, the raw counts of all samples were concatenated, yielding a total of 40 547 cells. The resulting dataset was normalized by the Seurat global-scaling normalization method ‘LogNormalize’ that normalizes the gene expression measurements for each cell by the total expression and multiplies it by a scale factor (10 000), and log-transforms the result. Highly variable genes were detected in Seurat according to the method described in Stuart et al. (73) and the data was scaled by linear transformation. Subsequently, the highly variable genes were used for principal component analysis (PCA). In order to remove batch effects and technical noise, the harmony package (v.1.0) was applied, using a theta parameter of 0. The resulting harmony-corrected PCA embeddings were used downstream for unsupervised Leiden clustering of the cells and UMAP dimensionality reduction, as implemented in Seurat.

## Data availability statement

The datasets presented in this study can be found in online repositories. The names of the repository/repositories and accession number(s) can be found below: GSE249611 (GEO).

## Ethics statement

The animal study was approved by the ethics committee of Ghent University, by the ethics committee of RIKEN Center for Brain Science and Nagoya City University, or by the ethics committee of Janssen Pharmaceutica. The study was conducted in accordance with the local legislation and institutional requirements.

## Author contributions

Gv: Conceptualization, Formal analysis, Funding acquisition, Investigation, Methodology, Project administration, Resources, Supervision, Validation, Visualization, Writing – original draft, Writing – review & editing. SS: Data curation, Formal analysis, Investigation, Methodology, Validation, Visualization, Writing – original draft, Writing – review & editing. DK: Data curation, Formal analysis, Investigation, Methodology, Software, Writing – review & editing. SD: Formal analysis, Investigation, Methodology, Writing – review & editing. TSS: Formal analysis, Investigation, Methodology, Writing – review & editing. MJ: Formal analysis, Investigation, Writing – review & editing. FB: Formal analysis, Investigation, Writing – review & editing. CV: Formal analysis, Investigation, Writing – review & editing. IP: Formal analysis, Investigation, Writing – review & editing. HM: Formal analysis, Investigation, Writing – review & editing. RV: Supervision, Writing – review & editing. EH: Formal analysis, Investigation, Writing – review & editing. SV: Formal analysis, Investigation, Writing – review & editing. IS: Data curation, Formal analysis, Investigation, Software, Writing – review & editing. BP: Data curation, Formal analysis, Investigation, Software, Writing – review & editing. SL: Supervision, Writing – review & editing. MS: Formal analysis, Investigation, Writing – review & editing. MP: Supervision, Writing – review & editing. TOS: Supervision, Writing – review & editing. AB: Conceptualization, Data curation, Formal analysis, Investigation, Methodology, Supervision, Validation, Writing – review & editing. KM: Data curation, Formal analysis, Investigation, Methodology, Software, Supervision, Writing – review & editing. ML: Conceptualization, Data curation, Formal analysis, Investigation, Methodology, Supervision, Validation, Writing – review & editing.

## Supplementary material

Supplementary Table 1 Summarizing table of experimental setup variables.

Supplementary Figure 1 Diagram outlining the null hypotheses and targeted nodes to test the role of inflammasome activation in ß-amyloid pathology. **(A)** Null hypothesis that microglia-selective A20 deletion induces Nlrp3 hyperactivation, neuroinflammation and exacerbation of amyloid pathology. **(B)** Null hypothesis that microglia-selective and full body deletion of caspase-1 prevents neuroinflammation and amyloid pathology. **(C)** Null hypothesis that full body Nlrp3 deletion prevents neuroinflammation and amyloid pathology.

Supplementary Figure 2 Schematic for generation of A20^FL^ and A20^Cx3Cr1-KO^ animals on *App^NL-G-F^* and *App^WT^* background. **(A)** Microglia gene targeting approach. **(B)** Breeding scheme. **(C)** Schematic for tamoxifen injection and experimental timepoints **(D)** Immunoblot for A20 expression on ex-vivo FACS-sorted microglia from control (A20^FL^) and A20^Cx3Cr1-KO^ mice 35 weeks after TAM injection. Actin shown as loading control. **(E)** Immunoblot for A20 expression on lysates from primary microglia from control (A20^FL^) and A20^Cx3Cr1-KO^ mice after stimulation with 4-OH-TAM. Actin is shown as loading control. Data are representative of two independent experiments.

Supplementary Figure 3 Microglial A20 deficiency enhances inflammatory gene expression independent of AD background. **(A)** Expression of NF-κB-associated pro-inflammatory mediators in hippocampal brain tissue of 20 week-old A20^FL^ and A20^Cx3Cr1-KO^ *App^WT^* and *App^NL-G-F^* animals. Each symbol represents one mouse, n=3 per group (*App^WT^*); n=8 per group (*App^NL-G-F^*). Data are presented as the ratio of the mRNA normalized to housekeeping genes and expressed as mean ± SEM. Significant differences were determined by a one-way ANOVA and multiple comparisons (*, p<0.05; **, p<0.001; ***, p<0.001; ****, p<0.0001). **(B)** Expression of NF-κB-associated pro-inflammatory mediators in hippocampal brain tissue of 56 week-old A20^FLand^ A20^Cx3Cr1-KO^ *App^WT^* and *App^NL-G-F^* animals. Each symbol represents one mouse, n=6 per group (*App^WT^*); n=13 per group (*App^NL-G-F^*). Data are presented as the ratio of the mRNA normalized to housekeeping genes and expressed as mean ± SEM. Significant differences were determined by a one-way ANOVA and multiple comparisons (*, p<0.05; ***, p<0.001; ****, p<0.0001).

Supplementary Figure 4 Microglial A20 deficiency does not exacerbate amyloid pathology in *App^NL-G-F^* mice. **(A)** Immunohistochemistry for 6E10^+^ amyloid plaque load in the whole brain of 20 week-old A20^FL^ and A20^Cx3Cr1-KO^ *App^WT^* and *App^NL-G-F^* mice. Scale bars: 50 µm (inset: 10µm). Representative images are displayed. **(B)** Average area (in µm^2^) of 6E10^+^ amyloid plaques across the whole brain of 20 week-old A20^FL^ (black) and A20^Cx3Cr1-KO^ (red) *App^NL-G-F^* mice. **(C)** Average number of 6E10^+^ amyloid plaques across the whole brain of 20 week-old A20^FL^ and A20^Cx3Cr1-KO^ *App^NL-G-F^* mice. **(D)** Quantification of 6E10^+^ amyloid pathology across the hippocampus of 20 week-old A20^FL^ and A20^Cx3Cr1-KO^ *App^NL-G-F^* mice. **(E)** Quantification of 6E10^+^ amyloid pathology across cortical regions (visual, posterior parietal association, somatosensory, somatomotor, orbital cortices; fiber tracts excluded) of 20 week-old A20^FL^ and A20^Cx3Cr1-KO^ *App^NL-G-F^* mice. **(F)** Quantification of brain-wide N25^+^ amyloid pathology of 20 week-old A20^FL^ and A20^Cx3Cr1-KO^ *App^NL-G-F^* mice. **(G)** Immunohistochemistry for 6E10^+^ amyloid plaque load in the whole brain of 56 week-old A20^FL^ and A20^Cx3Cr1-KO^ *App^WT^* and *App^NL-G-F^* mice. Scale bars: 50 µm (inset: 10µm). Representative images are displayed. **(H)** Average area (in µm^2^) of 6E10^+^ amyloid plaques across the whole brain of 56 week-old A20^FL^ (black) and A20^Cx3Cr1-KO^ (red) *App^NL-G-F^* mice. **(I)** Average number of 6E10^+^ amyloid plaques across the whole brain of 56 week-old A20^FL^ and A20^Cx3Cr1-KO^ *App^NL-G-F^* mice. **(J)** Quantification of 6E10^+^ amyloid pathology across the hippocampus of 56 week-old A20^FL^ and A20^Cx3Cr1-KO^ *App^NL-G-F^* mice. **(K)** Quantification of 6E10^+^ amyloid pathology across cortical regions (visual, posterior parietal association, somatosensory, somatomotor, orbital cortices; fiber tracts excluded) of 56 week-old A20^FL^ and A20^Cx3Cr1-KO^ *App^NL-G-F^* mice. **(L)** Quantification of brain-wide N25^+^ amyloid pathology of 56 week-old A20^FL^ and A20^Cx3Cr1-KO^ *App^NL-G-F^* mice. Each symbol represents one mouse (males, dark color; females, pale color). Data are represented as mean ± SEM. Significant differences are determined using two-way ANOVA (*, p<0.05).

Supplementary Figure 5 Microglial A20 deficiency does not exacerbate amyloid pathology in *App^NL-G-F^* mice. **(A)** Immunohistochemistry for Iba1^+^ microglia in the frontal cortex of 20 week-old A20^FL^ and A20^Cx3Cr1-KO^ *App^WT^* and *App^NL-G-F^* mice. Scale bars: 100µm (inset: 50 µm). Representative images are displayed. **(B)** Quantification of the number of Iba1^+^ microglia in cortex (visual, posterior parietal association, somatosensory, somatomotor, orbital cortices; fiber tracts excluded) of 20 week-old A20^FL^ (black) and A20^Cx3Cr1-KO^ (red) *App^WT^* and *App^NL-G-F^* mice. **(C)** Immunohistochemistry for Iba1^+^ microglia in the frontal cortex of 56 week-old A20^FL^ and A20^Cx3Cr1-KO^ *App^WT^* and *App^NL-G-F^* mice. Scale bars: 100µm (inset: 50 µm). Representative images are displayed. **(D)** Quantification of the number of Iba1^+^ microglia in cortex (visual, posterior parietal association, somatosensory, somatomotor, orbital cortices; fiber tracts excluded) of 56 week-old A20^FL^ (black) and A20^Cx3Cr1-KO^ (red) *App^WT^* and *App^NL-G-F^* mice. **(E)** Immunohistochemistry for GFAP^+^ astrocytes in the frontal cortex of 20 week-old A20^FL^ and A20^Cx3Cr1-KO^ *App^WT^* and *App^NL-G-F^* mice. Scale bars: 100µm (inset: 50 µm). Representative images are displayed. **(F)** Quantification of area covered by GFAP^+^ astrocytes in whole brain of 20 week-old A20^FL^ and A20^Cx3Cr1-KO^ *App^WT^* and *App^NL-G-F^* mice. **(G)** Immunohistochemistry for GFAP^+^ astrocytes in the frontal cortex of 56 week-old A20^FL^ and A20^Cx3Cr1-KO^ *App^WT^* and *App^NL-G-F^* mice. Scale bars: 100µm (inset: 50 µm). Representative images are displayed. **(H)** Quantification of area covered by GFAP^+^ astrocytes in whole brain of 56 week-old A20^FLand^ A20^Cx3Cr1-KO^ *App^WT^* and *App^NL-G-F^* mice. **(I)** Quantification of the number of plaque-associated dystrophic neurites (N25^+^ sAPP^+^), measured as percentage colocalization area, in whole brains of 20 week-old A20^FL^ and A20^Cx3Cr1-KO^ *App^NL-G-F^* mice. **(J)** Quantification of the number of plaque-associated dystrophic neurites (N25^+^ sAPP^+^), measured as percentage colocalization area, in whole brains of 56 week-old A20^FL^ and A20^Cx3Cr1-KO^ *App^NL-G-F^* mice. Each symbol represents one mouse (males, dark color; females, pale color). Data are represented as mean ± SEM. Significant differences are determined using two-way ANOVA (**, p<0.01; ***, p<0.001).

Supplementary Figure 6 Schematic for generation of casp1^FL^ and casp1^Cx3Cr1-KO^ animals on *App^NL-G-F^* and *App^WT^* background. **(A)** Microglia gene targeting approach. **(B)** Breeding scheme. **(C)** Schematic for tamoxifen injection and experimental timepoints. **(D)** Immunoblot for full-length casp1 expression in *ex-vivo* FACS-sorted microglia from control (casp1^FL^) and casp1^Cx3Cr1-KO^ 40 weeks after TAM injection. Actin shown as loading control. **(E)** Immunoblot for full-length casp1 expression in primary microglia lysates isolated from 0-3 day old casp1^FL^ and casp1^Cx3Cr1-KO^ neonates and stimulated with 4-OH TAM. Actin used as loading control.

Supplementary Figure 7 Microglial caspase-1 deficiency does not suppress AD pathology in 20 week-old *App^NL-G-F^* mice. **(A)** Immunohistochemistry for 6E10^+^ amyloid plaques in cortices of Casp1^FL^ and Casp1^Cx3Cr1-KO^ *App^WT^* and *App^NL-G-F^* mice. Scale bars: 100 µm (inset: 50 µm). Representative images are displayed. **(B)** Average area (in µm^2^) of 6E10^+^ amyloid plaques across the whole brain of 20 week-old Casp1^FL^ (black) and Casp1^Cx3Cr1-KO^ (blue) *App^NL-G-F^* mice. **(C)** Average number of 6E10^+^ amyloid plaques across the whole brain of 20 week-old Casp1^FL^ (black) and Casp1^Cx3Cr1-KO^ (blue) *App^NL-G-F^* mice. **(D)** Quantification of 6E10^+^ amyloid pathology across the hippocampus of 20 week-old Casp1^FL^ and Casp1^Cx3Cr1-KO^ *App^NL-G-F^* mice. **(E)** Quantification of 6E10^+^ amyloid pathology across cortical regions (visual, posterior parietal association, somatosensory, somatomotor, orbital cortices; fiber tracts excluded) of 20 week-old Casp1^FL^ and Casp1^Cx3Cr1-KO^ *App^NL-G-F^* mice. **(F)** Quantification of brain-wide N25^+^ amyloid pathology in 20 week-old Casp1^FL^ and Casp1^Cx3Cr1-KO^ *App^NL-G-F^* mice. **(G)** Immunohistochemistry for Iba1^+^ microglia in the cortex of 20 week-old Casp1^FL^ and Casp1^Cx3Cr1-KO^ *App^WT^* and *App^NL-G-F^* mice. Scale bars: 100µm (inset = 50 µm). Representative images are displayed. **(H)** Quantification of the number of Iba1^+^ microglia in cortices (visual, posterior parietal association, somatosensory, somatomotor, orbital cortices; fiber tracts excluded) of 20 week-old Casp1^FL^ and Casp1^Cx3Cr1-KO^ *App^WT^* and *App^NL-G-F^* mice. **(I)** Immunohistochemistry for GFAP^+^ astrocytes in the whole brain of 20 week-old Casp1^FL^ and Casp1^Cx3Cr1-KO^ *App^WT^* and *App^NL-G-F^* mice. Scale bars: 100µm (inset = 50 µm) Representative images are displayed. **(J)** Quantification of area covered by GFAP^+^ astrocytes in whole brain of 20 week-old Casp1^FL^ and Casp1^Cx3Cr1-KO^ *App^WT^* and *App^NL-G-F^* mice. **(K)** Quantification of the number of plaque-associated dystrophic neurites (N25^+^ sAPP^+^), measured as percentage colocalization area, in whole brains of 20 week-old Casp1^FL^ and Casp1^Cx3Cr1-KO^ *App^NL-G-F^* mice. Each symbol represents one mouse (males, dark color; females, pale color). Data are represented as mean ± SEM. Significant differences are determined using two-way ANOVA (****, p<0.0001).

Supplementary Figure 8 Microglial caspase-1 deficiency does not suppress AD pathology in 40 week-old *App^NL-G-F^* mice. **(A)** Immunohistochemistry for 6E10^+^ amyloid plaques in cortices of 40 week-old Casp1^FL^ and Casp1^Cx3Cr1-KO^ *App^NL-G-F^* mice. Scale bars: 100 µm (inset: 50 µm). Representative images are displayed. **(B)** Average area (in µm^2^) of 6E10^+^ amyloid plaques across the whole brain of 40 week-old Casp1^FL^ (black) and Casp1^Cx3Cr1-KO^ *App^NL-G-F^* (blue) mice. **(C)** Average number of 6E10^+^ amyloid plaques across the whole brain of 40 week-old Casp1^FL^ (black) and Casp1^Cx3Cr1-KO^ (blue) *App^NL-G-F^* mice. **(D)** Quantification of 6E10^+^ amyloid pathology across the hippocampus of 40 week-old Casp1^FL^ and Casp1^Cx3Cr1-KO^ *App^NL-G-F^* mice. **(E)** Quantification of 6E10^+^ amyloid pathology across cortical regions (visual, posterior parietal association, somatosensory, somatomotor, orbital cortices; fiber tracts excluded) of 40 week-old Casp1^FL^ and Casp1^Cx3Cr1-KO^ *App^NL-G-F^* mice. **(F)** Quantification of brain-wide N25^+^ amyloid pathology of 40 week-old Casp1^FL^ and Casp1^Cx3Cr1-KO^ *App^NL-G-F^* mice. **(G)** Immunohistochemistry for Iba1^+^ microglia in the cortex of 40 week-old Casp1^FL^ and Casp1^Cx3Cr1-KO^ *App^NL-G-F^* mice. Scale bars: 100µm (inset = 50 µm). Representative images are displayed. **(H)** Quantification of the number of Iba1^+^ microglia in cortices (visual, posterior parietal association, somatosensory, somatomotor, orbital cortices; fiber tracts excluded) of 40 week-old Casp1^FL^ and Casp1^Cx3Cr1-KO^ *App^NL-G-F^* mice. **(I)** Immunohistochemistry for GFAP^+^ astrocytes in the whole brain of 40 week-old Casp1^FL^ and Casp1^Cx3Cr1-KO^ *App^NL-G-F^* mice. Scale bars: 100µm (inset = 50 µm). Representative images are displayed. **(J)** Quantification of area covered by GFAP^+^ astrocytes in whole brain of 40 week-old Casp1^FL^ and Casp1^Cx3Cr1-KO^ *App^NL-G-F^* mice. **(K)** Quantification of the number of plaque-associated dystrophic neurites (N25^+^ sAPP^+^), measured as percentage colocalization area, in whole brains of 40 week-old Casp1^FL^ and Casp1^Cx3Cr1-KO^ *App^NL-G-F^* mice. Each symbol represents one mouse (males, dark color; females, pale color). Data are represented as mean ± SEM. **(L)** Quantification of the number of plaque-associated microglia (TMEM19^+^ Iba^+^) in 40 week-old casp1^FL^ and casp1^Cx3Cr1-KO^ *App^NL-G-F^*. Each symbol represents one mouse. Data are represented as mean ± SEM. **(M)** Immunohistochemistry for TMEM19^+^ Iba^+^ microglia in brain parenchyma of 40 week-old casp1^FL^ and casp1^Cx3Cr1-KO^ *App^NL-G-F^* mice. Scale bars: 50 µm. Asterisks indicate parenchymal non-plaque-associated cells; arrows show plaque-associated double-positive cells and arrowheads indicate plaque-associated Iba+TMEM- cells. **(N)** Mean GuHCl-extractable Aβ42 levels in (left) hippocampal and (right) cortical brain tissue. Each symbol represents one mouse, n=6 per group. Data are represented as mean ± SEM.

Supplementary Figure 9 Full-body Nlrp3 deficiency does not suppress ß-amyloid pathology in *APP/PS1* mice. **(A)** Quantification of 4G8+ amyloid plaque load in the cortex of 4 month-old Nlrp3^WT^ and Nlrp3^KO^ *APP/PS1* mice. Each symbol represents one mouse, n=14-23 per group. Data are represented as mean ± SEM. **(B)** Immunohistochemistry for 4G8+ amyloid plaque load in the cortex of 4 month-old Nlrp3^WT^ and Nlrp3^KO^ *APP/PS1* mice. Scale bars: 250 µm (inset: 50µm). Representative images are displayed. **(C)** Quantification of 4G8+ amyloid plaque load in the cortex of 6 month-old Nlrp3^WT^ and Nlrp3^KO^ *APP/PS1* mice. Each symbol represents one mouse, n=14-23 per group. Data are represented as mean ± SEM. **(D)** Immunohistochemistry for 4G8+ amyloid plaque load in the cortex of 4 month-old Nlrp3^WT^ and Nlrp3^KO^ *APP/PS1* mice. Scale bars: 250 µm (inset: 50µm). Representative images are displayed. **(E)** Quantification of 4G8+ amyloid plaque load in the cortex of 10 month-old Nlrp3^WT^ and Nlrp3^KO^ *APP/PS1* mice. Each symbol represents one mouse, n=14-23 per group. Data are represented as mean ± SEM. **(F)** Immunohistochemistry for 4G8+ amyloid plaque load in the cortex of 10 month-old Nlrp3^WT^ and Nlrp3^KO^ *APP/PS1* mice. Scale bars: 250 µm (inset: 50µm). Representative images are displayed. **(G)** Quantification of the number of cortical Iba1+ microglia in the cortex of 4 month-old Nlrp3^WT^ and Nlrp3^KO^ *APP/PS1* mice. Each symbol represents one mouse, n=14-23 per group. Data are represented as mean ± SEM. Significant differences are determined with One-way-ANOVA using Sidak’s multiple comparisons test (***, p < 0.001; ****, p < 0.0001). **(H)** Immunohistochemistry for Iba-1+ microglia in the cortex of 4 month-old Nlrp3^WT^ and Nlrp3^KO^ *APP/PS1* mice. Scale bars: 250 µm (inset: 50µm). Representative images are displayed. **(I)** Quantification of the number of cortical Iba1+ microglia in the cortex of 6 month-old Nlrp3^WT^ and Nlrp3^KO^ *APP/PS1* mice. Each symbol represents one mouse, n=14-23 per group. Data are represented as mean ± SEM. Significant differences are determined with One-way-ANOVA using Sidak’s multiple comparisons test (****, p < 0.0001). **(J)** Immunohistochemistry for Iba-1+ microglia in the cortex of 6 month-old Nlrp3^WT^ and Nlrp3^KO^ *APP/PS1* mice. Scale bars: 250 µm (inset: 50µm). Representative images are displayed. **(K)** Quantification of the number of cortical Iba1+ microglia in the cortex of 10 month-old Nlrp3^WT^ and Nlrp3^KO^ *APP/PS1* mice. Each symbol represents one mouse, n=14-23 per group. Data are represented as mean ± SEM. Significant differences are determined with One-way-ANOVA using Sidak’s multiple comparisons test (****, p < 0.0001). **(L)** Immunohistochemistry for Iba-1+ microglia in the cortex of 10 month-old Nlrp3^WT^ and Nlrp3^KO^ *APP/PS1* mice. Scale bars: 250 µm (inset: 50µm). Representative images are displayed.

Supplementary Figure 10 Nlrp3 deficiency does not suppress neuronal loss or Aβ40/Aβ42 brain levels in *APP/PS1* mice. **(A)** Quantification of NeuN+ labeled neurons in the hippocampus of 10 month-old Nlrp3^WT^ and Nlrp3^KO^ *APP/PS1* mice. Each symbol represents one mouse, n=14-23 per group. Data are represented as mean ± SEM. Significant differences were determined using One-way-ANOVA using Sidak’s multiple comparisons test (*, p < 0.05; **, p < 0.01). **(B)** Immunohistochemistry for NeuN+ neurons in the hippocampus of 10 month-old Nlrp3^WT^ and Nlrp3^KO^ *APP/PS1* mice. Scale bars: 500 µm (inset: 50µm). Representative images are displayed. **(C)** Quantification of NeuN+ labeled neurons in the cortex of 10 month-old Nlrp3^WT^ and Nlrp3^KO^ *APP/PS1* mice. Each symbol represents one mouse, n=14-23 per group. Data are represented as mean ± SEM. Significant differences were determined using One-way-ANOVA using Sidak’s multiple comparisons test (*, p < 0.05; **, p < 0.01). **(D)** Immunohistochemistry for NeuN+ neurons in the cortex of 10 month-old Nlrp3^WT^ and Nlrp3^KO^ *APP/PS1* mice. Scale bars: 500 µm (inset: 50µm). Representative images are displayed. **(E)** Quantification of Aβ40 amyloid levels in brain tissue of 10 month-old Nlrp3^WT^ and Nlrp3^KO^ *APP/PS1* mice. Data are represented as mean ± SEM. **(F)** Quantification of Aβ42 amyloid levels in brain tissue of 10 month-old Nlrp3^WT^ and Nlrp3^KO^ *APP/PS1* mice. Data are represented as mean ± SEM.

Supplementary Figure 11 Nlrp3 signaling does not shape microglial activation during homeostasis or upon amyloid pathology in *APP/PS1* mice. **(A)** UMAP plot of microglia from whole brain tissue of *Nlrp3* ^+/+^ and *Nlrp3* ^-/-^ mice, split by genotype and age. **(B)** Pie charts visualizing the percentage of cells per cluster in each genotype and age group from a. **(C)** Volcano plot, showing differentially expressed genes between microglia (all clusters combined) from *Nlrp3* ^-/-^ *vs. Nlrp3* ^+/+^ mice (3 months old). **(D)** Volcano plot, showing differentially expressed genes between microglia (all clusters combined) from *Nlrp3* ^-/-^ *vs*. *Nlrp3* ^+/+^ mice (6 months old). **(E)** Volcano plot, showing differentially expressed genes between microglia (all clusters combined) from *Nlrp3* ^-/-^ *vs*. *Nlrp3* ^+/+^ mice (9 months old). In red are shown the genes with adjP<0.05 and abs(log2FC)>1.

Supplementary Figure 12 Nlrp3 signaling does not shape microglial activation during homeostasis or upon amyloid pathology in *APP/PS1* mice. **(A)** Volcano plot, showing differentially expressed genes between microglia (all clusters combined) from *Nlrp3* ^-/-^ *APP/PS1 vs. Nlrp3* ^+/+^ *APP/PS1* mice (3 months old). **(B)** UMAP plots, visualizing the expression level of key inflammasome-related genes in microglia from *Nlrp3* ^-/-^ *APP/PS1* and *Nlrp3* ^+/+^ *APP/PS1* mice.
