# Supplementary material for: Inflammasome signaling is dispensable for ß-amyloid-induced neuropathology in preclinical models of Alzheimer’s disease
Source: Front Immunol. 2024 Jan 29;15:1323409. doi: 10.3389/fimmu.2024.1323409 (PMC10863058; doi:10.3389/fimmu.2024.1323409)
Supplement: Supplementary Table 1 — Summarizing table of experimental setup variables. [file Table_1.docx]

**Supplementary Table 1 : summarizing table comparing the two studies**

|  | ***Srinivasan et al.*** | ***Heneka et al., 2013*** |
| --- | --- | --- |
| **Mouse line** | *App*^NL-G-F^ (Saito et al., 2014)  APP/PS1 (Radde *et al.*, 2006) | APP/PS1 (Jankowsky et al., 2001) |
| **Genetic background** | C57/Bl6 | C57/Bl6 |
| **Age** | *App*^NL-G-F^ : 20-40-56-70 weeks  APP/PS1 : 20-30-50-70 weeks | APP/PS1 : 16 months |
| **Gender** | Male and female mice | Not disclosed |
| **Mouse facility** | Housed in a Specific pathogen free (SPF) at 21 °C and a 14/10 h light/dark cycle with free access to food and water | Housed under standard conditions at 22 °C and a 12/12 h light/dark cycle with free access to food and water (health status not disclosed). |
| **Chow** | V1534 Ssniff R-M/H 10 mm mouse autoclavable | Not disclosed |
