# Supplementary figures and images for: Inflammasome signaling is dispensable for ß-amyloid-induced neuropathology in preclinical models of Alzheimer’s disease
Source: Front Immunol. 2024 Jan 29;15:1323409. doi: 10.3389/fimmu.2024.1323409 (PMC10863058; doi:10.3389/fimmu.2024.1323409)

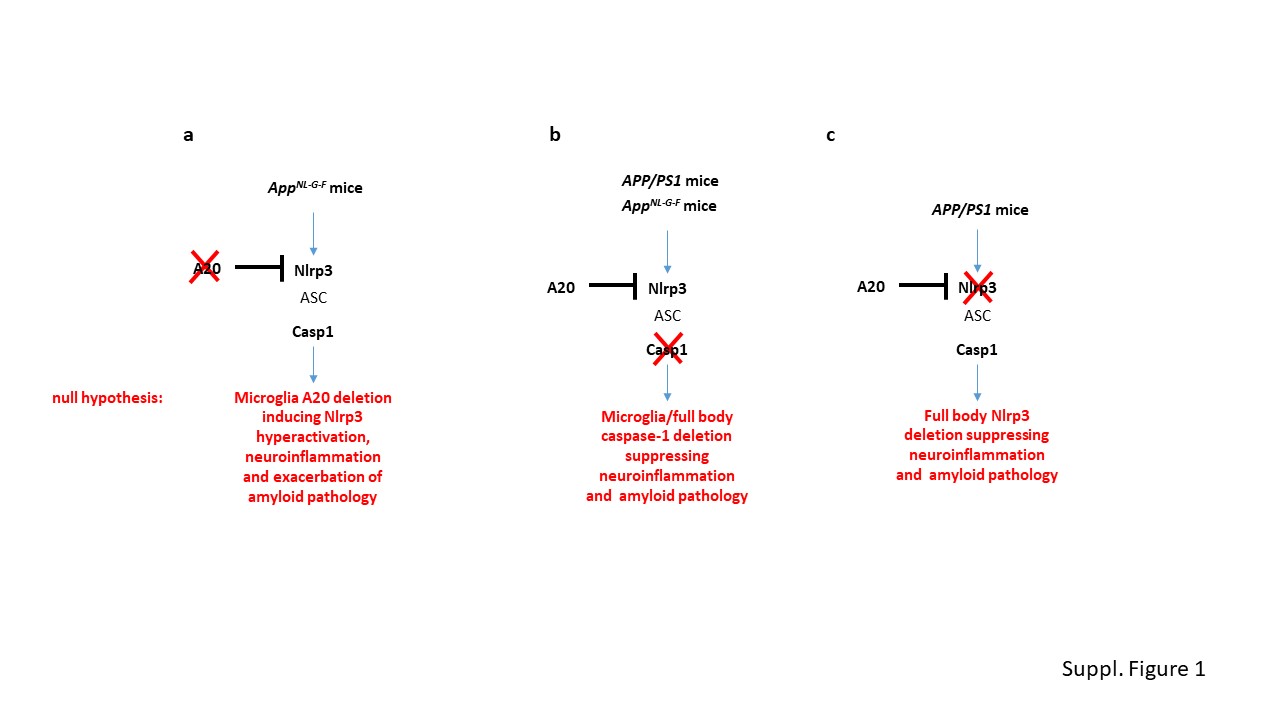

Supplement: Supplementary Figure 1 — Diagram outlining the null hypotheses and targeted nodes to test the role of inflammasome activation in ß-amyloid pathology. (A) Null hypothesis that microglia-selective A20 deletion induces Nlrp3 hyperactivation, neuroinflammation and exacerbation of amyloid pathology. (B) Null hypothesis that microglia-selective and full body deletion of caspase-1 prevents neuroinflammation and amyloid pathology. (C) Null hypothesis that full body Nlrp3 deletion prevents neuroinflammation and amyloid pathology. [file Image_1.jpg]

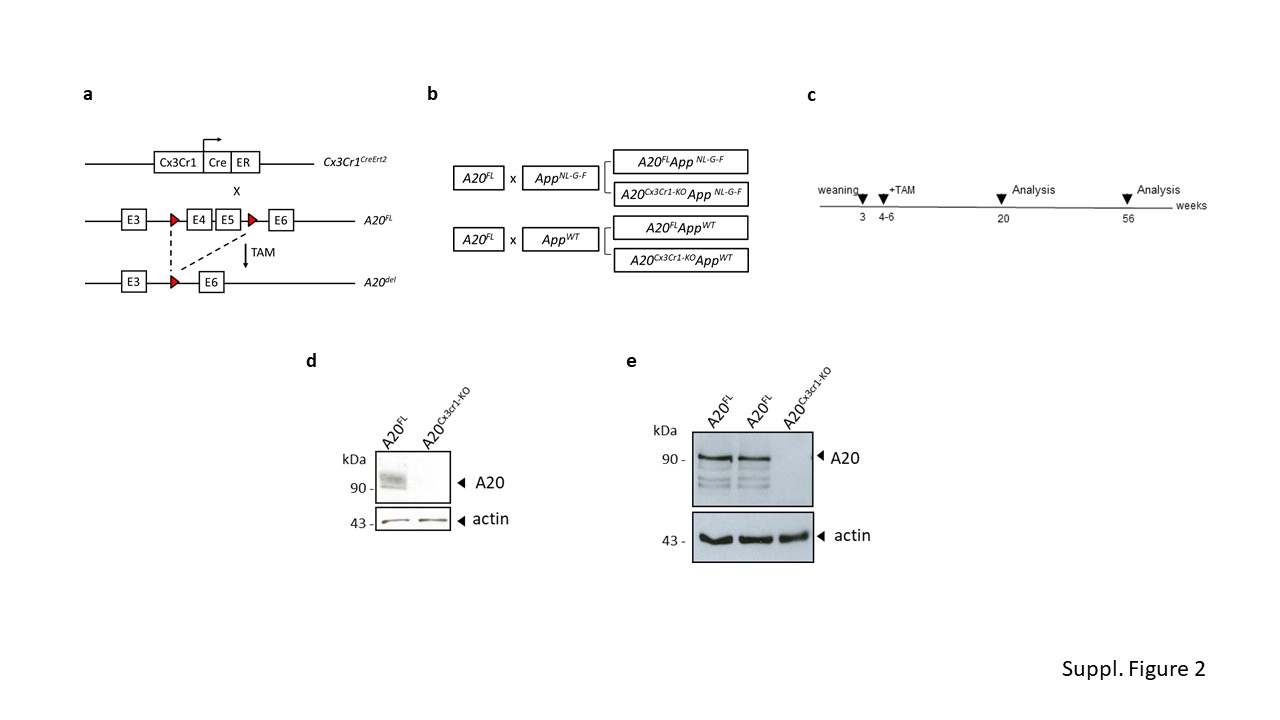

Supplement: Supplementary Figure 2 — Schematic for generation of A20FL and A20Cx3Cr1-KO animals on AppNL-G-F and AppWT background. (A) Microglia gene targeting approach. (B) Breeding scheme. (C) Schematic for tamoxifen injection and experimental timepoints (D) Immunoblot for A20 expression on ex-vivo FACS-sorted microglia from control (A20FL) and A20Cx3Cr1-KO mice 35 weeks after TAM injection. Actin shown as loading control. (E) Immunoblot for A20 expression on lysates from primary microglia from control (A20FL) and A20Cx3Cr1-KO mice after stimulation with 4-OH-TAM. Actin is shown as loading control. Data are representative of two independent experiments. [file Image_2.jpg]

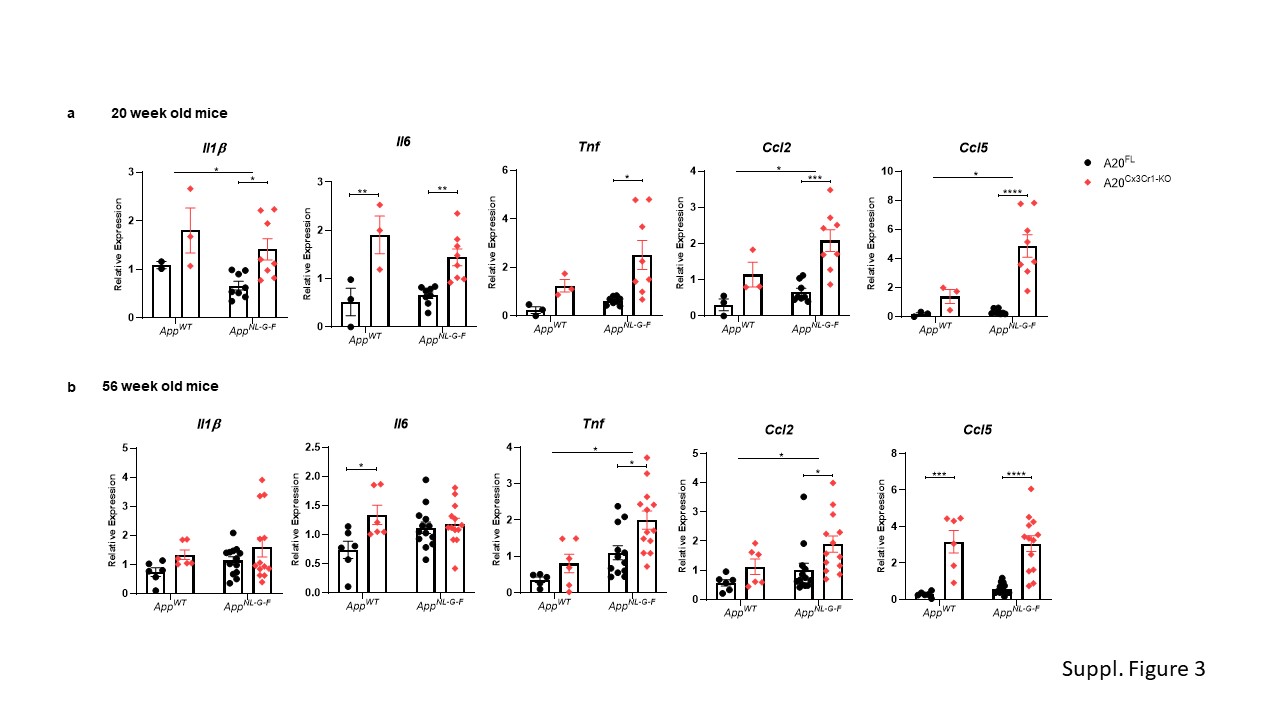

Supplement: Supplementary Figure 3 — Microglial A20 deficiency enhances inflammatory gene expression independent of AD background. (A) Expression of NF-κB-associated pro-inflammatory mediators in hippocampal brain tissue of 20 week-old A20FL and A20Cx3Cr1-KO AppWT and AppNL-G-F animals. Each symbol represents one mouse, n=3 per group (AppWT ); n=8 per group (AppNL-G-F ). Data are presented as the ratio of the mRNA normalized to housekeeping genes and expressed as mean ± SEM. Significant differences were determined by a one-way ANOVA and multiple comparisons (*, p<0.05; **, p<0.001; ***, p<0.001; ****, p<0.0001). (B) Expression of NF-κB-associated pro-inflammatory mediators in hippocampal brain tissue of 56 week-old A20FLand A20Cx3Cr1-KO AppWT and AppNL-G-F animals. Each symbol represents one mouse, n=6 per group (AppWT ); n=13 per group (AppNL-G-F ). Data are presented as the ratio of the mRNA normalized to housekeeping genes and expressed as mean ± SEM. Significant differences were determined by a one-way ANOVA and multiple comparisons (*, p<0.05; ***, p<0.001; ****, p<0.0001). [file Image_3.jpg]

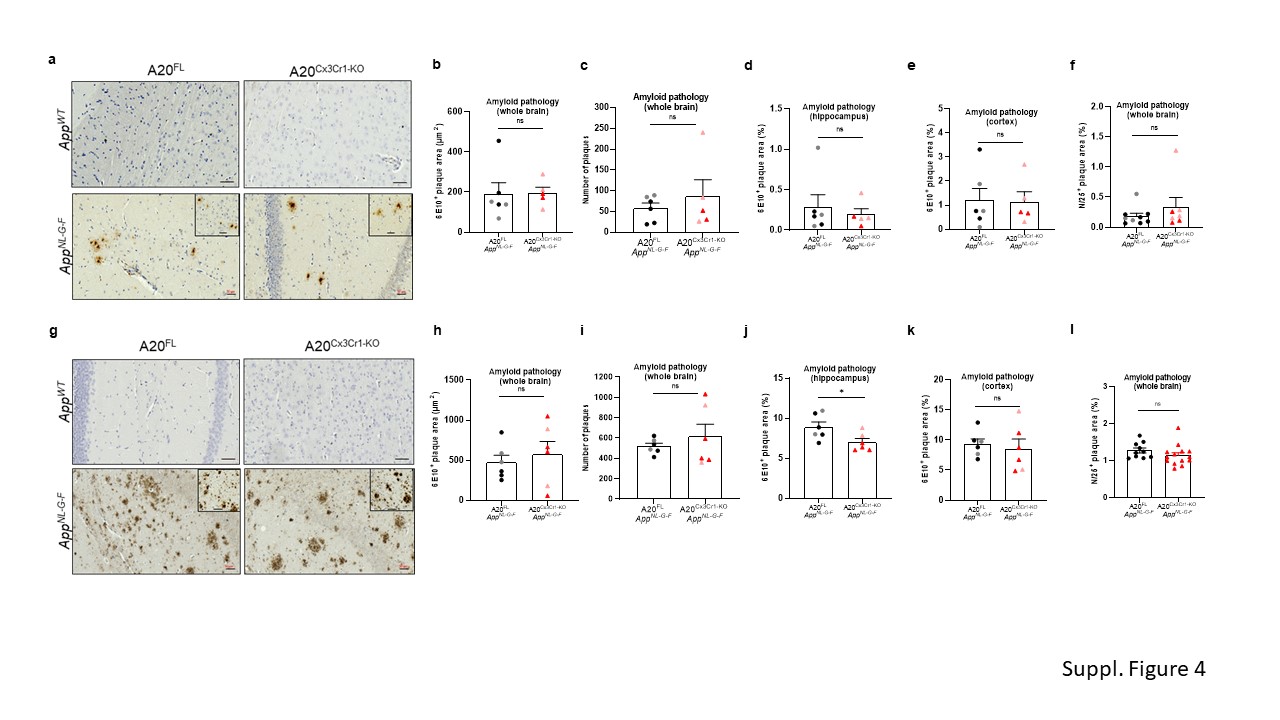

Supplement: Supplementary Figure 4 — Microglial A20 deficiency does not exacerbate amyloid pathology in AppNL-G-F mice. (A) Immunohistochemistry for 6E10+ amyloid plaque load in the whole brain of 20 week-old A20FL and A20Cx3Cr1-KO AppWT and AppNL-G-F mice. Scale bars: 50 µm (inset: 10µm). Representative images are displayed. (B) Average area (in µm2) of 6E10+ amyloid plaques across the whole brain of 20 week-old A20FL (black) and A20Cx3Cr1-KO (red) AppNL-G-F mice. (C) Average number of 6E10+ amyloid plaques across the whole brain of 20 week-old A20FL and A20Cx3Cr1-KO AppNL-G-F mice. (D) Quantification of 6E10+ amyloid pathology across the hippocampus of 20 week-old A20FL and A20Cx3Cr1-KO AppNL-G-F mice. (E) Quantification of 6E10+ amyloid pathology across cortical regions (visual, posterior parietal association, somatosensory, somatomotor, orbital cortices; fiber tracts excluded) of 20 week-old A20FL and A20Cx3Cr1-KO AppNL-G-F mice. (F) Quantification of brain-wide N25+ amyloid pathology of 20 week-old A20FL and A20Cx3Cr1-KO AppNL-G-F mice. (G) Immunohistochemistry for 6E10+ amyloid plaque load in the whole brain of 56 week-old A20FL and A20Cx3Cr1-KO AppWT and AppNL-G-F mice. Scale bars: 50 µm (inset: 10µm). Representative images are displayed. (H) Average area (in µm2) of 6E10+ amyloid plaques across the whole brain of 56 week-old A20FL (black) and A20Cx3Cr1-KO (red) AppNL-G-F mice. (I) Average number of 6E10+ amyloid plaques across the whole brain of 56 week-old A20FL and A20Cx3Cr1-KO AppNL-G-F mice. (J) Quantification of 6E10+ amyloid pathology across the hippocampus of 56 week-old A20FL and A20Cx3Cr1-KO AppNL-G-F mice. (K) Quantification of 6E10+ amyloid pathology across cortical regions (visual, posterior parietal association, somatosensory, somatomotor, orbital cortices; fiber tracts excluded) of 56 week-old A20FL and A20Cx3Cr1-KO AppNL-G-F mice. (L) Quantification of brain-wide N25+ amyloid pathology of 56 week-old A20FL and A20Cx3Cr1-KO AppNL-G-F mice. Each symbol represents one mouse (males, [file Image_4.jpg]

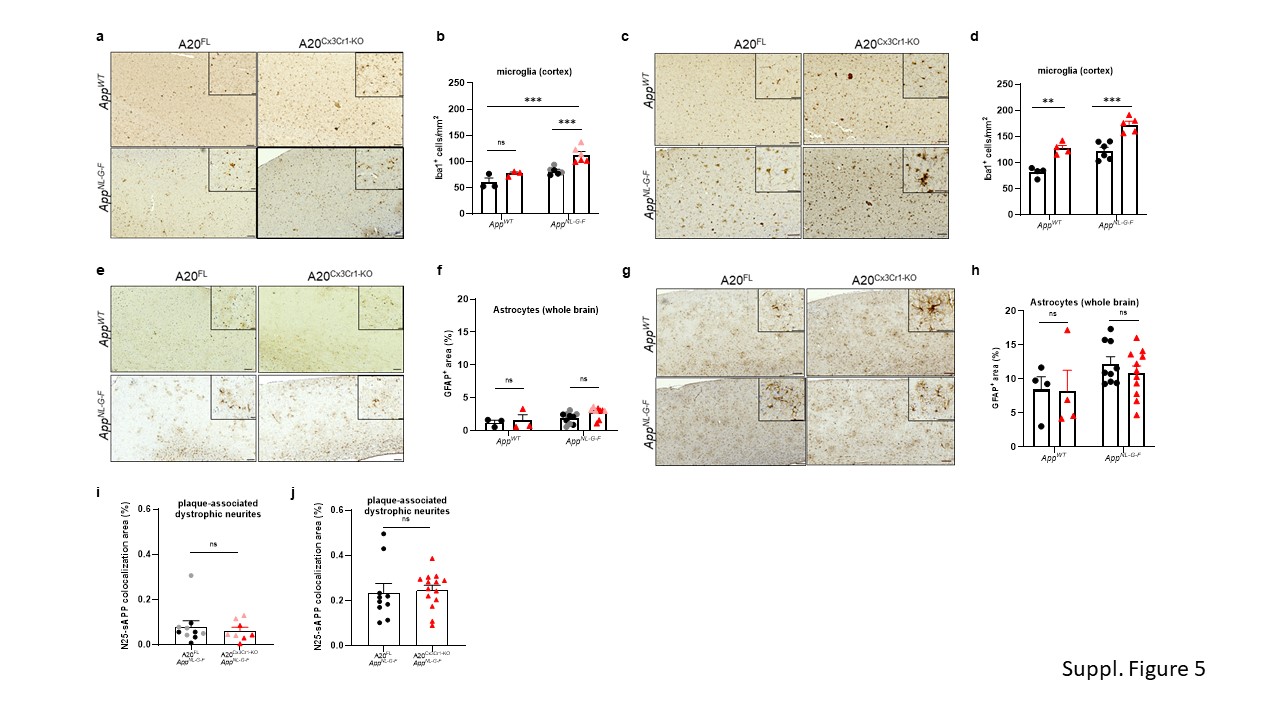

Supplement: Supplementary Figure 5 — Microglial A20 deficiency does not exacerbate amyloid pathology in AppNL-G-F mice. (A) Immunohistochemistry for Iba1+ microglia in the frontal cortex of 20 week-old A20FL and A20Cx3Cr1-KO AppWT and AppNL-G-F mice. Scale bars: 100µm (inset: 50 µm). Representative images are displayed. (B) Quantification of the number of Iba1+ microglia in cortex (visual, posterior parietal association, somatosensory, somatomotor, orbital cortices; fiber tracts excluded) of 20 week-old A20FL (black) and A20Cx3Cr1-KO (red) AppWT and AppNL-G-F mice. (C) Immunohistochemistry for Iba1+ microglia in the frontal cortex of 56 week-old A20FL and A20Cx3Cr1-KO AppWT and AppNL-G-F mice. Scale bars: 100µm (inset: 50 µm). Representative images are displayed. (D) Quantification of the number of Iba1+ microglia in cortex (visual, posterior parietal association, somatosensory, somatomotor, orbital cortices; fiber tracts excluded) of 56 week-old A20FL (black) and A20Cx3Cr1-KO (red) AppWT and AppNL-G-F mice. (E) Immunohistochemistry for GFAP+ astrocytes in the frontal cortex of 20 week-old A20FL and A20Cx3Cr1-KO AppWT and AppNL-G-F mice. Scale bars: 100µm (inset: 50 µm). Representative images are displayed. (F) Quantification of area covered by GFAP+ astrocytes in whole brain of 20 week-old A20FL and A20Cx3Cr1-KO AppWT and AppNL-G-F mice. (G) Immunohistochemistry for GFAP+ astrocytes in the frontal cortex of 56 week-old A20FL and A20Cx3Cr1-KO AppWT and AppNL-G-F mice. Scale bars: 100µm (inset: 50 µm). Representative images are displayed. (H) Quantification of area covered by GFAP+ astrocytes in whole brain of 56 week-old A20FLand A20Cx3Cr1-KO AppWT and AppNL-G-F mice. (I) Quantification of the number of plaque-associated dystrophic neurites (N25+ sAPP+), measured as percentage colocalization area, in whole brains of 20 week-old A20FL and A20Cx3Cr1-KO AppNL-G-F mice. (J) Quantification of the number of plaque-associated dystrophic neurites (N25+ sAPP+), measured as percentage colocalization area, in who [file Image_5.jpg]

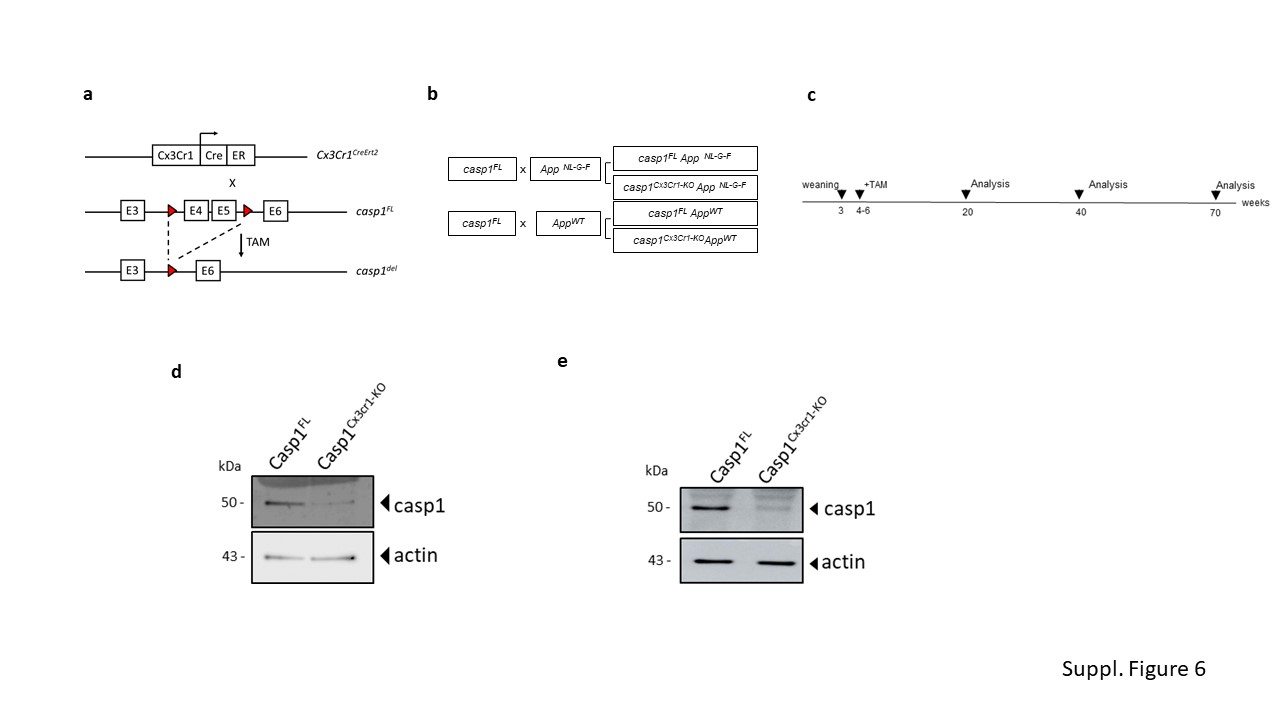

Supplement: Supplementary Figure 6 — Schematic for generation of casp1FL and casp1Cx3Cr1-KO animals on AppNL-G-F and AppWT background. (A) Microglia gene targeting approach. (B) Breeding scheme. (C) Schematic for tamoxifen injection and experimental timepoints. (D) Immunoblot for full-length casp1 expression in ex-vivo FACS-sorted microglia from control (casp1FL) and casp1Cx3Cr1-KO 40 weeks after TAM injection. Actin shown as loading control. (E) Immunoblot for full-length casp1 expression in primary microglia lysates isolated from 0-3 day old casp1FL and casp1Cx3Cr1-KO neonates and stimulated with 4-OH TAM. Actin used as loading control. [file Image_6.jpg]

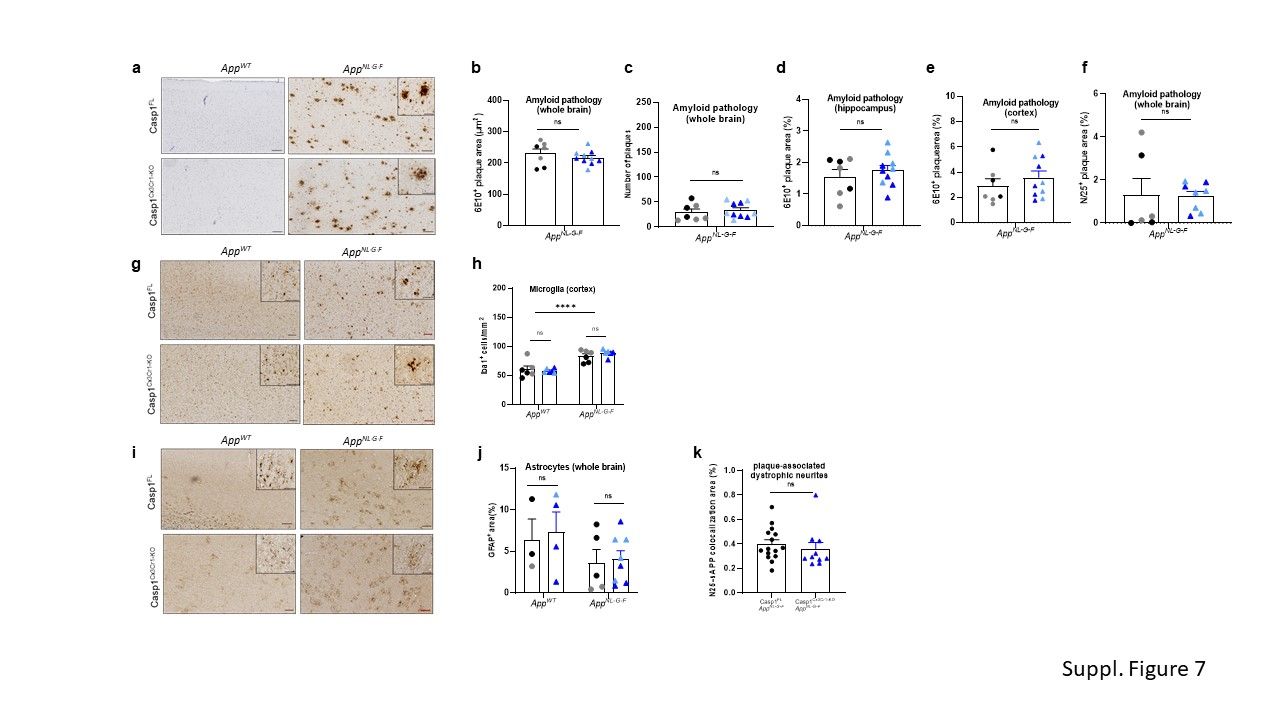

Supplement: Supplementary Figure 7 — Microglial caspase-1 deficiency does not suppress AD pathology in 20 week-old AppNL-G-F mice. (A) Immunohistochemistry for 6E10+ amyloid plaques in cortices of Casp1FL and Casp1Cx3Cr1-KO AppWT and AppNL-G-F mice. Scale bars: 100 µm (inset: 50 µm). Representative images are displayed. (B) Average area (in µm2) of 6E10+ amyloid plaques across the whole brain of 20 week-old Casp1FL (black) and Casp1Cx3Cr1-KO (blue) AppNL-G-F mice. (C) Average number of 6E10+ amyloid plaques across the whole brain of 20 week-old Casp1FL (black) and Casp1Cx3Cr1-KO (blue) AppNL-G-F mice. (D) Quantification of 6E10+ amyloid pathology across the hippocampus of 20 week-old Casp1FL and Casp1Cx3Cr1-KO AppNL-G-F mice. (E) Quantification of 6E10+ amyloid pathology across cortical regions (visual, posterior parietal association, somatosensory, somatomotor, orbital cortices; fiber tracts excluded) of 20 week-old Casp1FL and Casp1Cx3Cr1-KO AppNL-G-F mice. (F) Quantification of brain-wide N25+ amyloid pathology in 20 week-old Casp1FL and Casp1Cx3Cr1-KO AppNL-G-F mice. (G) Immunohistochemistry for Iba1+ microglia in the cortex of 20 week-old Casp1FL and Casp1Cx3Cr1-KO AppWT and AppNL-G-F mice. Scale bars: 100µm (inset = 50 µm). Representative images are displayed. (H) Quantification of the number of Iba1+ microglia in cortices (visual, posterior parietal association, somatosensory, somatomotor, orbital cortices; fiber tracts excluded) of 20 week-old Casp1FL and Casp1Cx3Cr1-KO AppWT and AppNL-G-F mice. (I) Immunohistochemistry for GFAP+ astrocytes in the whole brain of 20 week-old Casp1FL and Casp1Cx3Cr1-KO AppWT and AppNL-G-F mice. Scale bars: 100µm (inset = 50 µm) Representative images are displayed. (J) Quantification of area covered by GFAP+ astrocytes in whole brain of 20 week-old Casp1FL and Casp1Cx3Cr1-KO AppWT and AppNL-G-F mice. (K) Quantification of the number of plaque-associated dystrophic neurites (N25+ sAPP+), measured as percentage colocalization area, in whole brains of 20 week-old Cas [file Image_7.jpg]

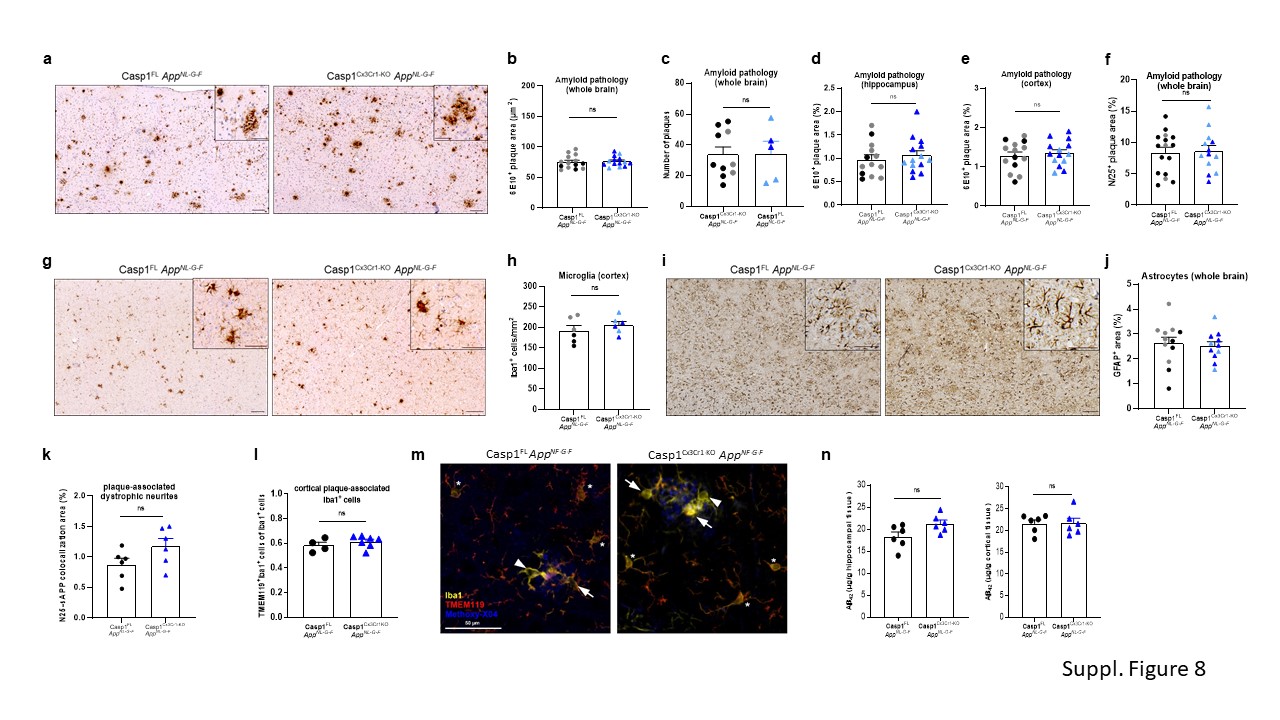

Supplement: Supplementary Figure 8 — Microglial caspase-1 deficiency does not suppress AD pathology in 40 week-old AppNL-G-F mice. (A) Immunohistochemistry for 6E10+ amyloid plaques in cortices of 40 week-old Casp1FL and Casp1Cx3Cr1-KO AppNL-G-F mice. Scale bars: 100 µm (inset: 50 µm). Representative images are displayed. (B) Average area (in µm2) of 6E10+ amyloid plaques across the whole brain of 40 week-old Casp1FL (black) and Casp1Cx3Cr1-KO AppNL-G-F (blue) mice. (C) Average number of 6E10+ amyloid plaques across the whole brain of 40 week-old Casp1FL (black) and Casp1Cx3Cr1-KO (blue) AppNL-G-F mice. (D) Quantification of 6E10+ amyloid pathology across the hippocampus of 40 week-old Casp1FL and Casp1Cx3Cr1-KO AppNL-G-F mice. (E) Quantification of 6E10+ amyloid pathology across cortical regions (visual, posterior parietal association, somatosensory, somatomotor, orbital cortices; fiber tracts excluded) of 40 week-old Casp1FL and Casp1Cx3Cr1-KO AppNL-G-F mice. (F) Quantification of brain-wide N25+ amyloid pathology of 40 week-old Casp1FL and Casp1Cx3Cr1-KO AppNL-G-F mice. (G) Immunohistochemistry for Iba1+ microglia in the cortex of 40 week-old Casp1FL and Casp1Cx3Cr1-KO AppNL-G-F mice. Scale bars: 100µm (inset = 50 µm). Representative images are displayed. (H) Quantification of the number of Iba1+ microglia in cortices (visual, posterior parietal association, somatosensory, somatomotor, orbital cortices; fiber tracts excluded) of 40 week-old Casp1FL and Casp1Cx3Cr1-KO AppNL-G-F mice. (I) Immunohistochemistry for GFAP+ astrocytes in the whole brain of 40 week-old Casp1FL and Casp1Cx3Cr1-KO AppNL-G-F mice. Scale bars: 100µm (inset = 50 µm). Representative images are displayed. (J) Quantification of area covered by GFAP+ astrocytes in whole brain of 40 week-old Casp1FL and Casp1Cx3Cr1-KO AppNL-G-F mice. (K) Quantification of the number of plaque-associated dystrophic neurites (N25+ sAPP+), measured as percentage colocalization area, in whole brains of 40 week-old Casp1FL and Casp1Cx3Cr1-KO AppNL-G-F mic [file Image_8.jpg]

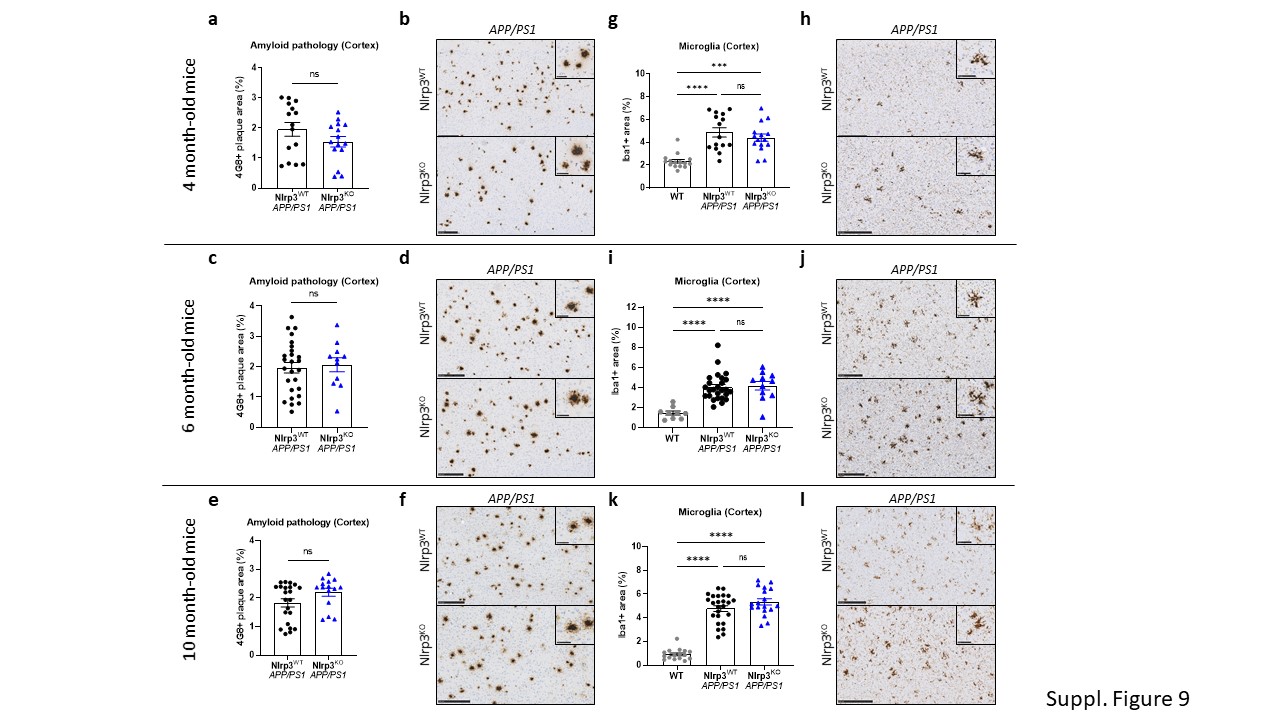

Supplement: Supplementary Figure 9 — Full-body Nlrp3 deficiency does not suppress ß-amyloid pathology in APP/PS1 mice. (A) Quantification of 4G8+ amyloid plaque load in the cortex of 4 month-old Nlrp3WT and Nlrp3KO APP/PS1 mice. Each symbol represents one mouse, n=14-23 per group. Data are represented as mean ± SEM. (B) Immunohistochemistry for 4G8+ amyloid plaque load in the cortex of 4 month-old Nlrp3WT and Nlrp3KO APP/PS1 mice. Scale bars: 250 µm (inset: 50µm). Representative images are displayed. (C) Quantification of 4G8+ amyloid plaque load in the cortex of 6 month-old Nlrp3WT and Nlrp3KO APP/PS1 mice. Each symbol represents one mouse, n=14-23 per group. Data are represented as mean ± SEM. (D) Immunohistochemistry for 4G8+ amyloid plaque load in the cortex of 4 month-old Nlrp3WT and Nlrp3KO APP/PS1 mice. Scale bars: 250 µm (inset: 50µm). Representative images are displayed. (E) Quantification of 4G8+ amyloid plaque load in the cortex of 10 month-old Nlrp3WT and Nlrp3KO APP/PS1 mice. Each symbol represents one mouse, n=14-23 per group. Data are represented as mean ± SEM. (F) Immunohistochemistry for 4G8+ amyloid plaque load in the cortex of 10 month-old Nlrp3WT and Nlrp3KO APP/PS1 mice. Scale bars: 250 µm (inset: 50µm). Representative images are displayed. (G) Quantification of the number of cortical Iba1+ microglia in the cortex of 4 month-old Nlrp3WT and Nlrp3KO APP/PS1 mice. Each symbol represents one mouse, n=14-23 per group. Data are represented as mean ± SEM. Significant differences are determined with One-way-ANOVA using Sidak’s multiple comparisons test (***, p < 0.001; ****, p < 0.0001). (H) Immunohistochemistry for Iba-1+ microglia in the cortex of 4 month-old Nlrp3WT and Nlrp3KO APP/PS1 mice. Scale bars: 250 µm (inset: 50µm). Representative images are displayed. (I) Quantification of the number of cortical Iba1+ microglia in the cortex of 6 month-old Nlrp3WT and Nlrp3KO APP/PS1 mice. Each symbol represents one mouse, n=14-23 per group. Data are represented as mean ± SEM. Significant dif [file Image_9.jpg]

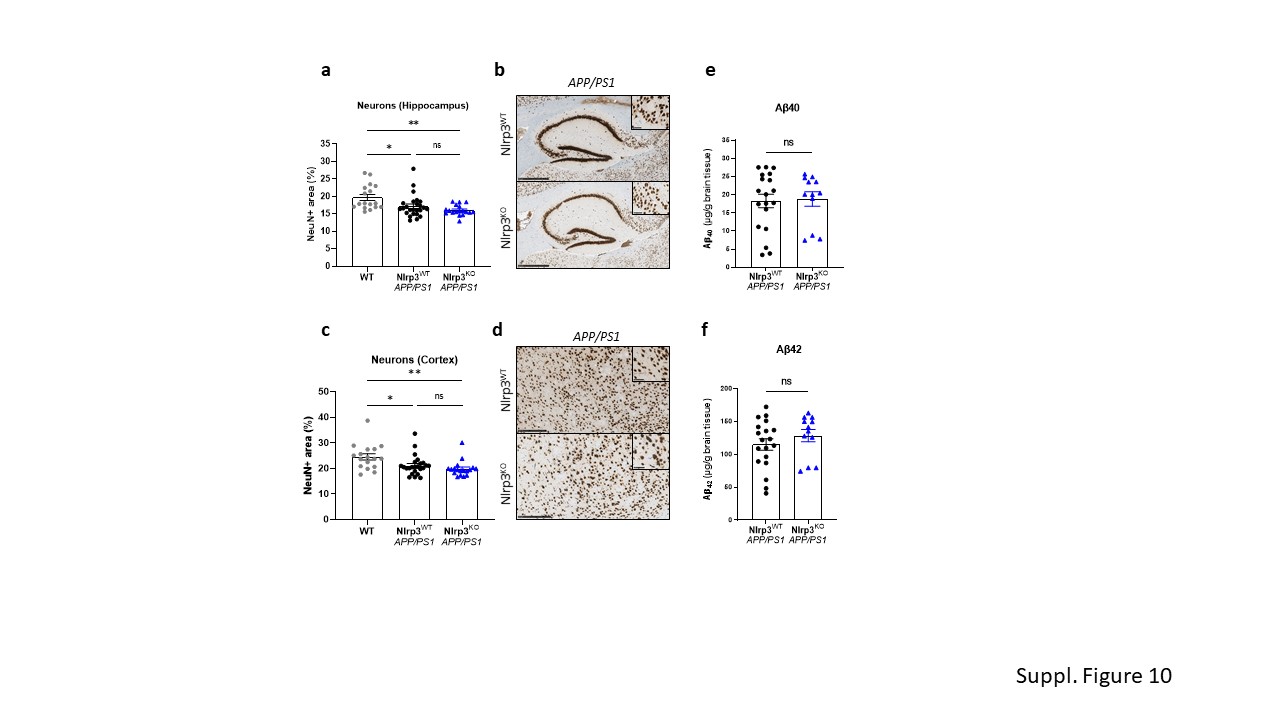

Supplement: Supplementary Figure 10 — Nlrp3 deficiency does not suppress neuronal loss or Aβ40/Aβ42 brain levels in APP/PS1 mice. (A) Quantification of NeuN+ labeled neurons in the hippocampus of 10 month-old Nlrp3WT and Nlrp3KO APP/PS1 mice. Each symbol represents one mouse, n=14-23 per group. Data are represented as mean ± SEM. Significant differences were determined using One-way-ANOVA using Sidak’s multiple comparisons test (*, p < 0.05; **, p < 0.01). (B) Immunohistochemistry for NeuN+ neurons in the hippocampus of 10 month-old Nlrp3WT and Nlrp3KO APP/PS1 mice. Scale bars: 500 µm (inset: 50µm). Representative images are displayed. (C) Quantification of NeuN+ labeled neurons in the cortex of 10 month-old Nlrp3WT and Nlrp3KO APP/PS1 mice. Each symbol represents one mouse, n=14-23 per group. Data are represented as mean ± SEM. Significant differences were determined using One-way-ANOVA using Sidak’s multiple comparisons test (*, p < 0.05; **, p < 0.01). (D) Immunohistochemistry for NeuN+ neurons in the cortex of 10 month-old Nlrp3WT and Nlrp3KO APP/PS1 mice. Scale bars: 500 µm (inset: 50µm). Representative images are displayed. (E) Quantification of Aβ40 amyloid levels in brain tissue of 10 month-old Nlrp3WT and Nlrp3KO APP/PS1 mice. Data are represented as mean ± SEM. (F) Quantification of Aβ42 amyloid levels in brain tissue of 10 month-old Nlrp3WT and Nlrp3KO APP/PS1 mice. Data are represented as mean ± SEM. [file Image_10.jpg]

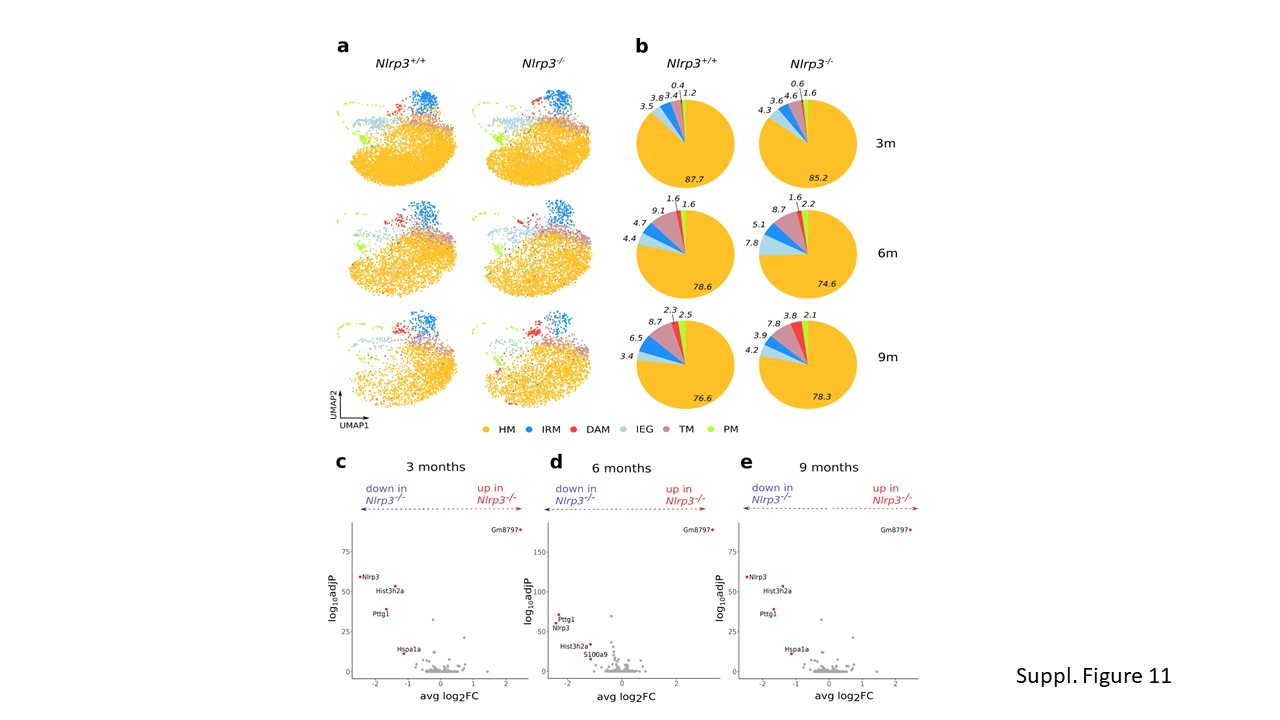

Supplement: Supplementary Figure 11 — Nlrp3 signaling does not shape microglial activation during homeostasis or upon amyloid pathology in APP/PS1 mice. (A) UMAP plot of microglia from whole brain tissue of Nlrp3 +/+ and Nlrp3 -/- mice, split by genotype and age. (B) Pie charts visualizing the percentage of cells per cluster in each genotype and age group from a. (C) Volcano plot, showing differentially expressed genes between microglia (all clusters combined) from Nlrp3 -/- vs. Nlrp3 +/+ mice (3 months old). (D) Volcano plot, showing differentially expressed genes between microglia (all clusters combined) from Nlrp3 -/- vs. Nlrp3 +/+ mice (6 months old). (E) Volcano plot, showing differentially expressed genes between microglia (all clusters combined) from Nlrp3 -/- vs. Nlrp3 +/+ mice (9 months old). In red are shown the genes with adjP<0.05 and abs(log2FC)>1. [file Image_11.jpg]

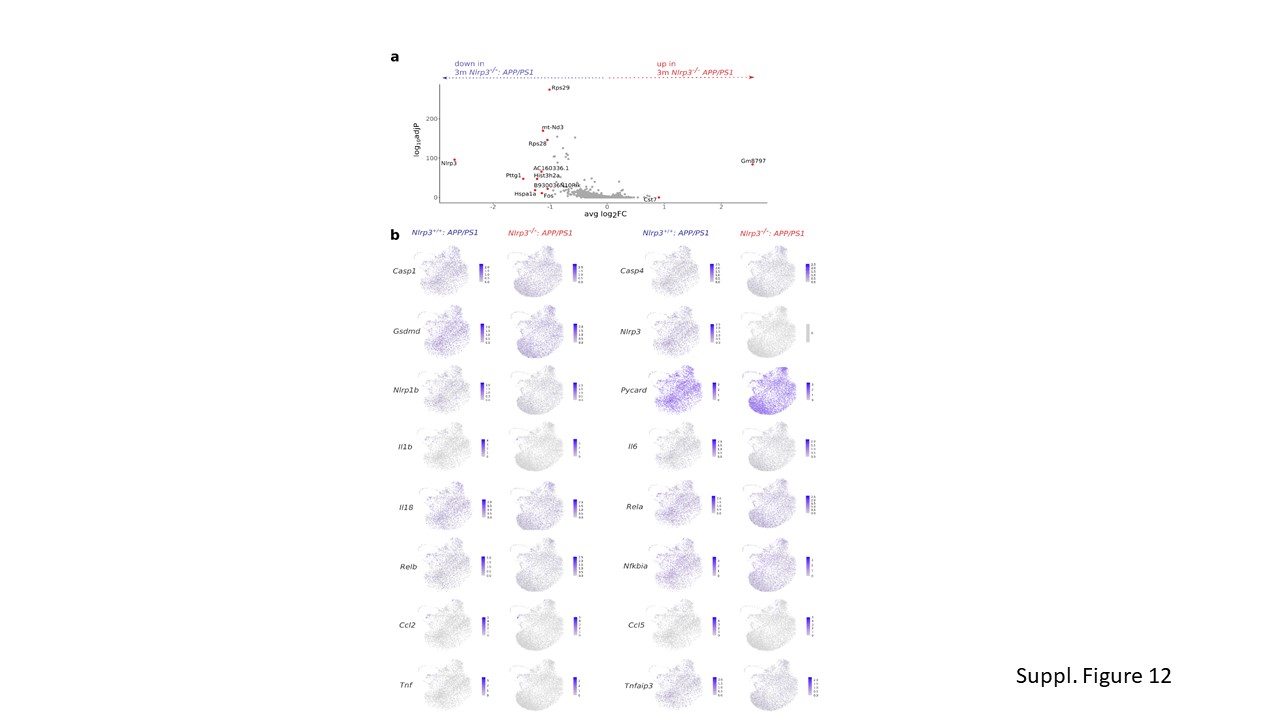

Supplement: Supplementary Figure 12 — Nlrp3 signaling does not shape microglial activation during homeostasis or upon amyloid pathology in APP/PS1 mice. (A) Volcano plot, showing differentially expressed genes between microglia (all clusters combined) from Nlrp3 -/- APP/PS1 vs. Nlrp3 +/+ APP/PS1 mice (3 months old). (B) UMAP plots, visualizing the expression level of key inflammasome-related genes in microglia from Nlrp3 -/- APP/PS1 and Nlrp3 +/+ APP/PS1 mice. [file Image_12.jpg]
